# Supplementary material for: Mass spectrometry reveals the evolutionary conservation of phycobiliprotein complexes
Source: Nat Commun. 2026 Feb 16;17:2834. doi: 10.1038/s41467-026-69558-y (PMC13022049; doi:10.1038/s41467-026-69558-y)
Supplement: Supplementary file 1 — Supplementary Information [file 41467_2026_69558_MOESM1_ESM.pdf]

Supplementary Information

Mass spectrometry reveals the evolutionary conservation of phycobiliprotein complexes.

Jaspreet K. Sound[1], Giorgio Bianchini[2], Thrupthi A. Ashok[1], Cecilia Rad-Menéndez[3,4], David H. Green[3], Patricia Sánchez-Baracaldo[2], Aneika C. Leney[1]*

Supporting Methods.

DNA extraction.

DNA extraction for P. priestleyi ANT.L61.2 was performed using a Qiagen DNeasy Ultraclean Microbial (12224-50) extraction kit with an optimized procedure for DNA extraction. CCAP strains were extracted using DNeasy Plant Mini kit essentially as outlined by the manufacturer (Qiagen). The extracted DNA was quantified and assayed using an Implen N60 NanoPhotometer, a Qubit HS dsDNA kit (Invitrogen Q32851) on an Invitrogen Qubit 3 Fluorometer (Q33216), and gel electrophoresis. The DNA was stored at -20 °C.

Genome sequencing and assembly.

The genome for CCAP strain 1479/10 was obtained from GenBank under the Whole Genome Shotgun accession JAFKRJ000000000 (1).

For P. priestleyi ANT.L61.2, genome sequencing was performed at the Centre for Genomic Research of the University of Liverpool. CCAP strains were sequenced by Novogene UK following their PCR-free shotgun metagenomic Illumina library preparation protocol and paired-end (PE150) sequenced on NovaSeq 6000. An Illumina fragment library was prepared using the NEBNext Ultra II FS Kit (1/2 volume reactions), targeting ~350 bp inserts. Paired-end sequencing (2 x 150 bp) was done with an Illumina NovaSeq using SP chemistry. Illumina adapters were removed from the raw Fastq files using Cutadapt v1.2.1(2), with the option -O 3. The reads were further trimmed using Sickle v1.2(3) with a minimum window quality score of 20 and removing reads shorter than 15 bp. CCAP metagenomic data were pre-processed using metaWRAP read_qc module(4) with default settings.

The trimmed read files were provided by the sequencing center and analyzed using FastQC v0.11.9(5); no further trimming was deemed necessary. The genome was assembled following an update to a previously implemented approach(6–8). The genome was initially assembled using SPAdes v3.15.2(9) with a read coverage cutoff value of 20; four different combinations of k-mer sizes were used (67,77,87,97; 21,33,55,77; 21,33,55,61,71,81,91,101,111,121; 21,33,55,61,71,81,91,101,111,121,127), in combination with the --isolate or the --careful options (8 assemblies in total). The resulting assemblies were analyzed using QUAST v5.2.0(10), and the values for multiple statistics (N50, L50, N90, auN, number of contigs, largest contig) were inspected; the de Bruijn graphs were then visualized using Bandage v0.8.1(11). The best assembly (careful mode with k-

mer sizes 21,33,55,61,71,81,91,101,111,121,127) was selected, and the others were discarded. CCAP strains were assembled using metaWRAP assembly module and SPAdes v3.13.3(9) (discarding contigs <1000 bp).

As the strains were grown in unialgal, but not axenic, conditions, it was necessary to isolate the cyanobacterial genome and remove contaminants. This was performed in two different ways. First, for strain ANT.L61.2, BUSCO v5.4.3(12) was used to search for 773 cyanobacterial single copy orthologs (cyanobacteria_odb10 lineage) in the assembly and rnammer v1.2(13) was used to predict 16S small subunit ribosomal RNA sequences. Furthermore, each node of the de Bruijn graph produced by SPAdes was divided into three sections, each containing respectively the first 1000 nucleotides, the last 1000 nucleotides, and the central 1000 nucleotides of the node; blastn v2.11.0+(14) was then used to perform a database search of each node section against a local copy of the NCBI nt database(15). The taxonomic assignment of the best hits for each section was automatically analyzed to classify the nodes as probably cyanobacterial, non-cyanobacterial, chimeric, or undetermined (nodes shorter than 1000 nucleotides were treated as a single entity). The de Bruijn graph was then visualized in Bandage, and the genes identified by BUSCO were mapped to the graph nodes using the built-in tblastn search function, with 90% Identity and 10-10 e-value filters; nodes were colored according to their BLAST taxonomic classification. For nodes with uncertain classification, the BLAST search was repeated manually against the NCBI nt database, using the Web BLAST interface(16). The second genome assembly strategy as used by CCAP was as follows: the metaWRAP pipeline(4) was used for read quality assessment and genome assembly using SPAdes v3.13 (metagenomic mode), followed by ensemble genome binning using Concoct(17), MaxBin2(18) and MetaBat2(19) and CheckM v1(20). Ensemble bins were polished using metaWRAP reassemble bins module. The metagenome assembled genomes were verified using CheckM(20), taxonomically identified with GTDB-Tk(21) and annotated using Prokka (v1.14.6)(22).

Non-cyanobacterial nodes were identified by manually inspecting the Bandage plot (Figure S16), and scaffolds containing them were removed from the assembly. SSPACE Standard v3.0(23) was used to extend the “cleaned” assembly, and Bowtie v2.4.5(24) was used to map the original reads to the assembly. Samtools v1.13-30-ga78376c(25) was used to extract the reads that were mapped to the assembly, and then the entire genome assembly process was repeated using only these reads. Scaffolds shorter than 200 bp were removed, and BUSCO was used with the cyanobacteria_odb10

lineage to assess the completeness of the final assembly (99.6%); CheckM v1.2.2(20) was used to assess the contamination level (1.65%). The assembly consisted of 266 scaffolds, for a total of 6.4 Mbp, with 48.79% GC content (N50 = 66621, L50 = 30, auN = 78745.6, as determined by QUAST). The genome was submitted to JGI IMG/ER(26) for annotation (GOLD Analysis Project ID: Ga0610072). This Whole Genome Shotgun project has been deposited at DDBJ/ENA/GenBank under the accession JBHLFI000000000. The version described in this paper is version JBHLFI010000000. The reads as provided by the sequencing center have been deposited in the Sequence Read Archive under the BioProject PRJNA1163361. The MAG genome data for the CCAP strains and the SAMS 01UC strain have been deposited at GenBank under the BioProject number PRJNA1127564.

UV-visible absorbance spectroscopy.

Absorbance spectroscopy related to Figure S26 was performed on phycobiliprotein extracts in 100 mM ammonium acetate pH 6.8 either alone or when mixed in a 1:1 ratio with a phycobiliprotein extract from another species. Absorbance was measured on a Jenway 7315 Spectrophotometer over 200-800 nm range taking readings at 2 nm intervals. Measurements were performed in triplicate with the average of three replicates reported. All spectroscopy data were baseline corrected and normalized to the absorbance reading at 280 nm.

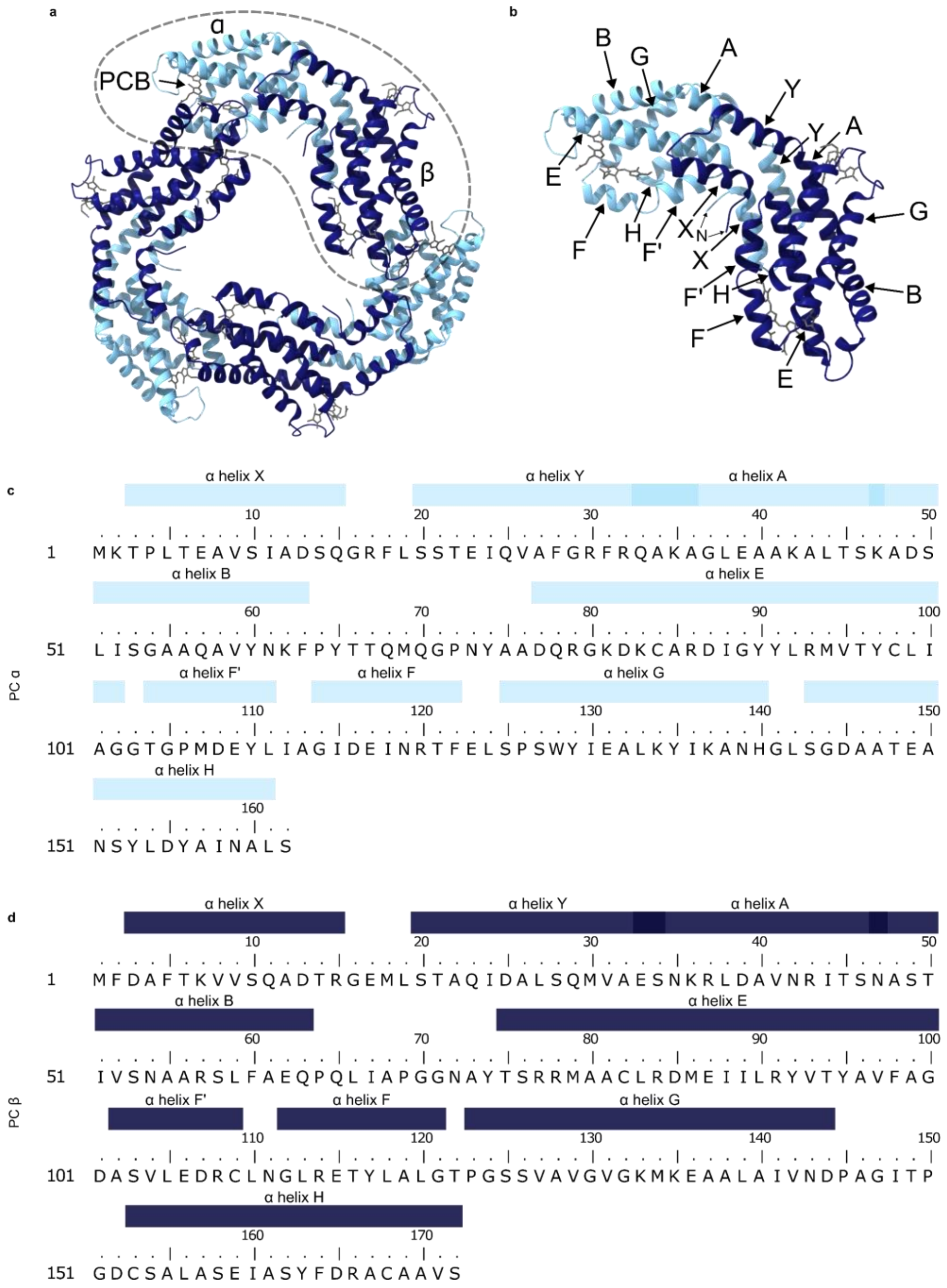

Fig S1. Structure of Phycocyanin. Hexameric structure of phycocyanin (a, PDB:1HA7(37)) alongside the annotated α-helices within the individual α and β subunits of the dimeric phycocyanin building block (b). Location of these α-helices are plotted onto the sequence of the α (c) and β subunits (d).

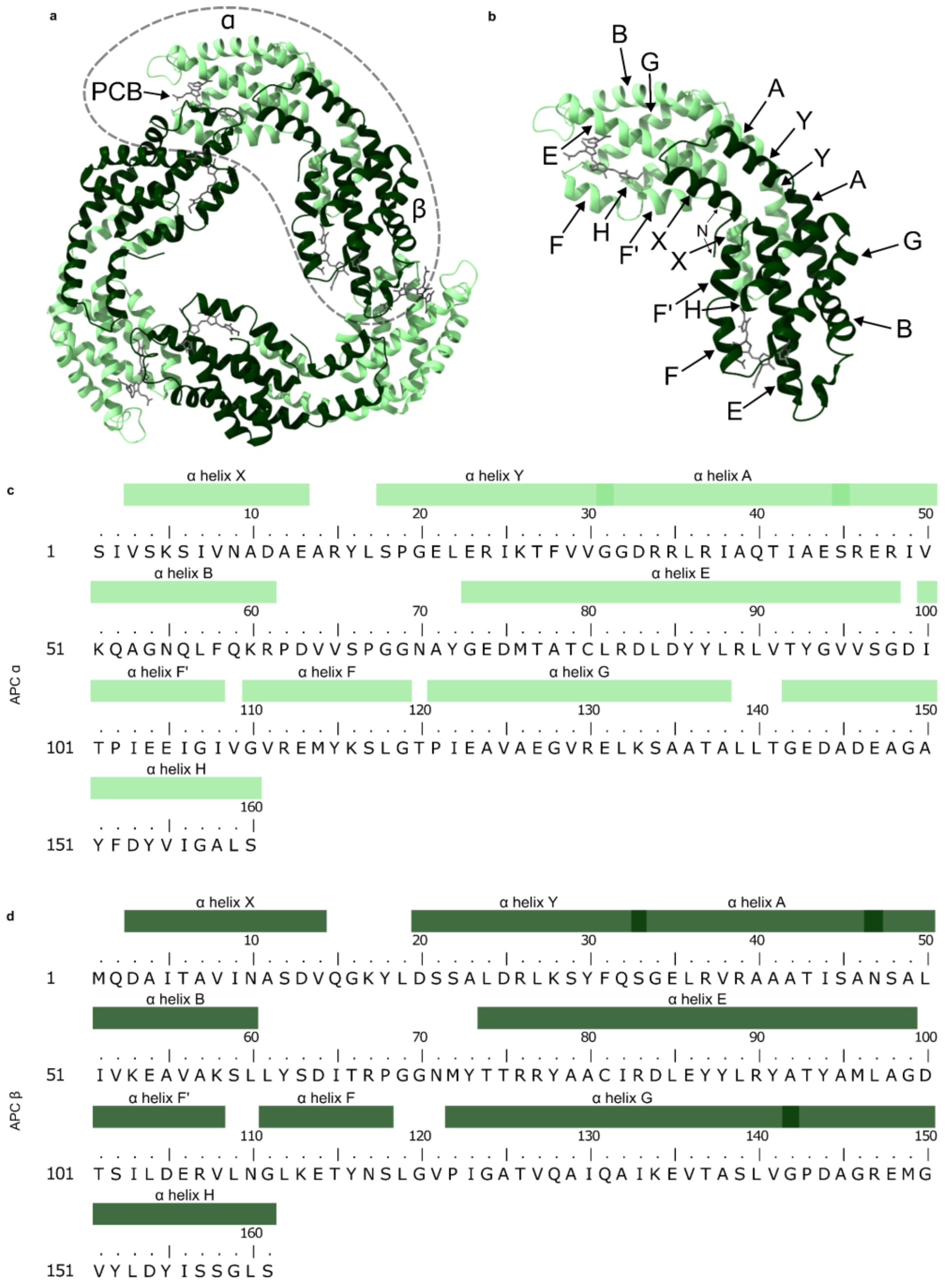

Fig S2: Structure of Allophycocyanin. Hexameric structure of allophycocyanin (a, PDB:4F0U(38)) alongside the annotated α-helices within the individual α and β subunits of the dimeric allophycocyanin building block (b). Location of these α-helices are plotted onto the sequence of the α (c) and β subunits (d).

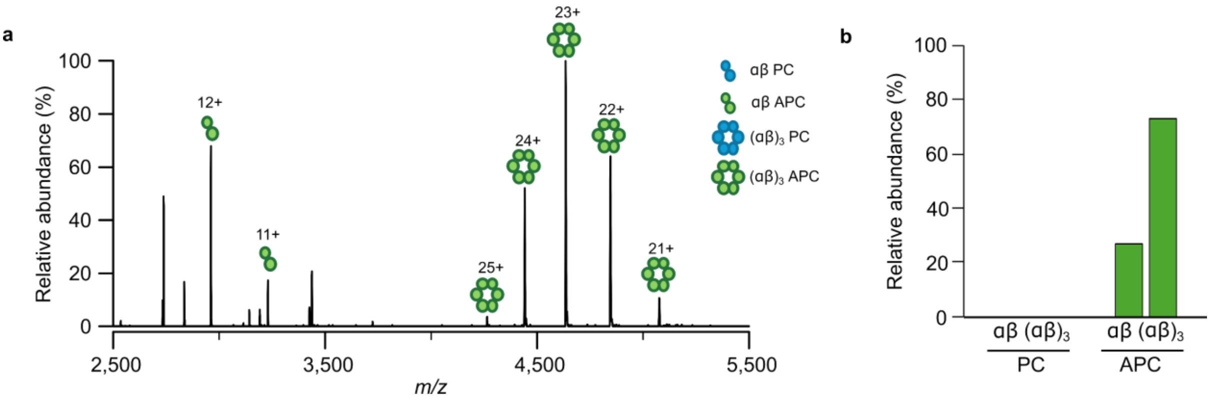

Fig S3. Native mass spectrum of phycobiliprotein extract from *Nostoc muscorum* (CCAP 1453/12) (a) highlighting the relative abundance of the allophycocyanin (APC) complexes detected (b). Note that phycocyanin was of low abundance within this cell lysate thus its oligomeric status could not be quantified.

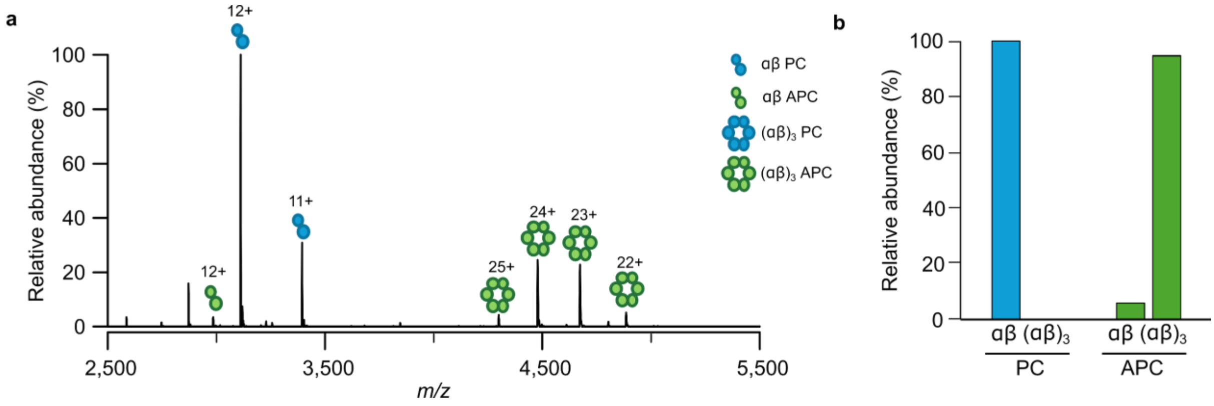

Fig S4. Native mass spectrum of phycobiliprotein extract from *Kamptonema sp.* (SAMS 01UC) (a) highlighting the relative abundance of the allophycocyanin (APC) and phycocyanin (PC) complexes detected (b).

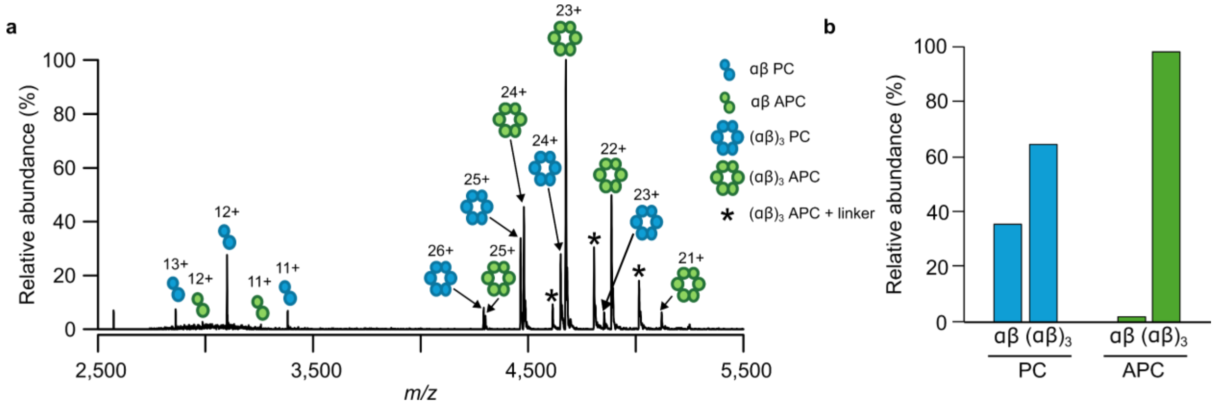

Fig S5. Native mass spectrum of phycobiliprotein extract from *Phormidesmis priestleyi* (ANT.L61.2) (a) highlighting the relative abundance of the allophycocyanin (APC) and phycocyanin (PC) complexes detected (b). The * indicates the APC complex with linker protein (ApcC) bound.

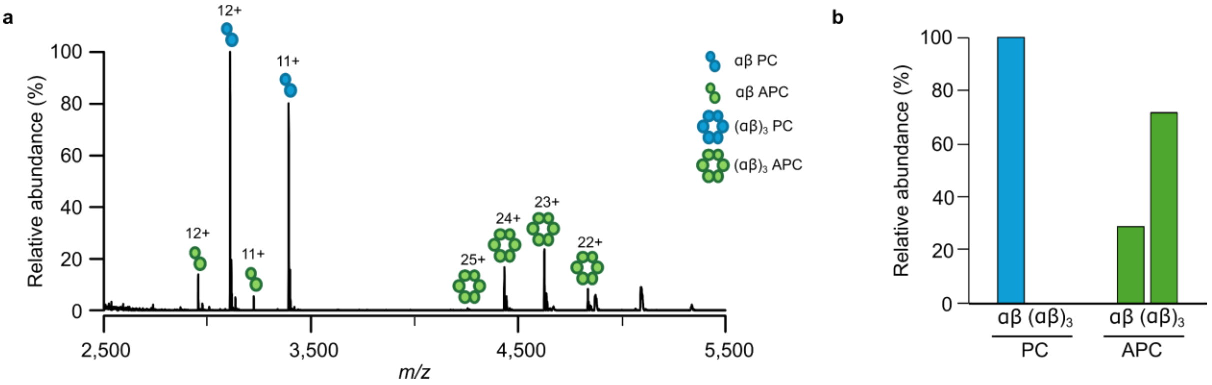

Fig S6. Native mass spectrum of phycobiliprotein extract from *Spirulina major* (CCAP 1475/3) (a) highlighting the relative abundance of the allophycocyanin (APC) and phycocyanin (PC) complexes detected (b).

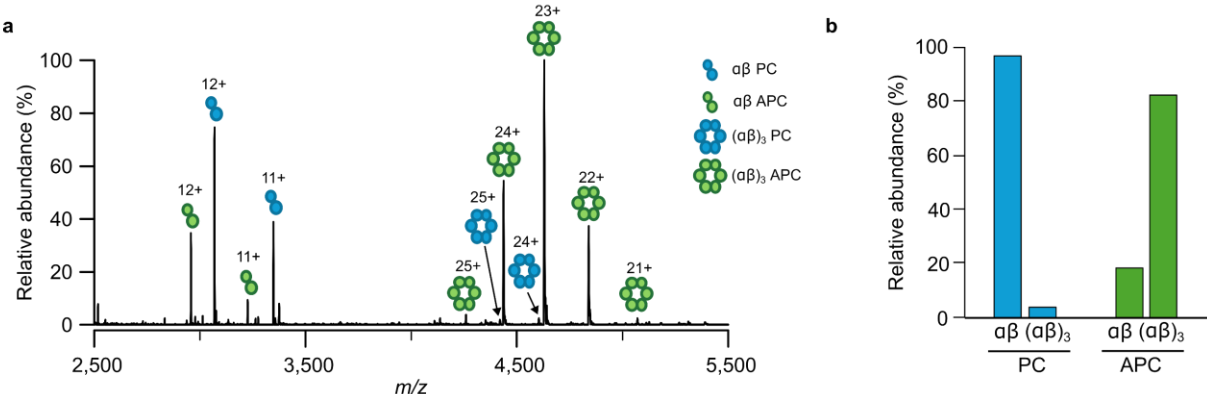

Fig S7. Native mass spectrum of phycobiliprotein extract from *Dolichospermum circinale* (CCAP 1403/21) (a) highlighting the relative abundance of the allophycocyanin (APC) and phycocyanin (PC) complexes detected (b).

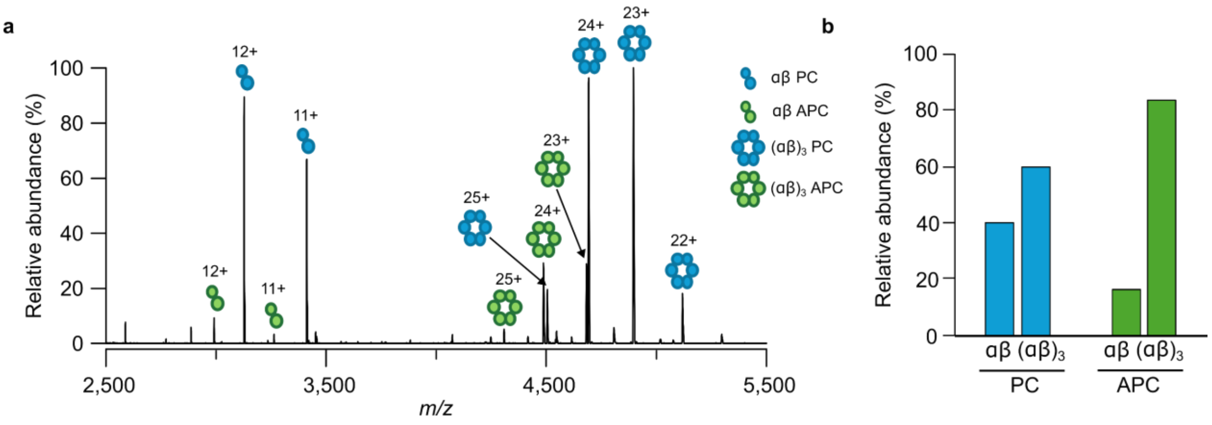

Fig S8. Native mass spectrum of phycobiliprotein extract from *Gloeomargarita lithophora* (CCAP 1437/1) (a) highlighting the relative abundance of the allophycocyanin (APC) and phycocyanin (PC) complexes detected (b).

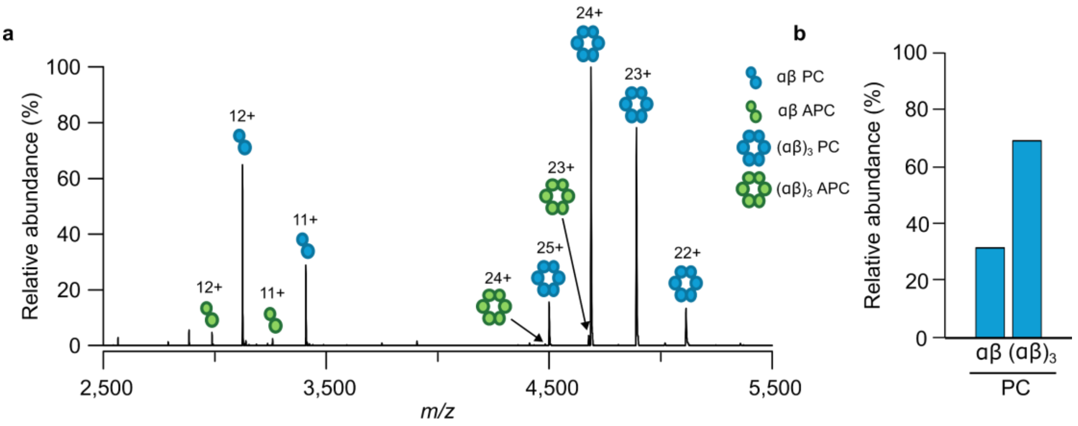

Fig S9. Native mass spectrum of phycobiliprotein extract from *Synechococcus sp.* (CCAP 1479/10) (a) highlighting the relative abundance of the allophycocyanin (APC) and phycocyanin (PC) complexes detected (b). Note that the 22+ charge state of the $(\alpha\beta)_3$ APC complex overlaps with the 23+ charge state of the $(\alpha\beta)_3$ PC complex, therefore, the dimer:hexamer ratio in this case was not quantified.

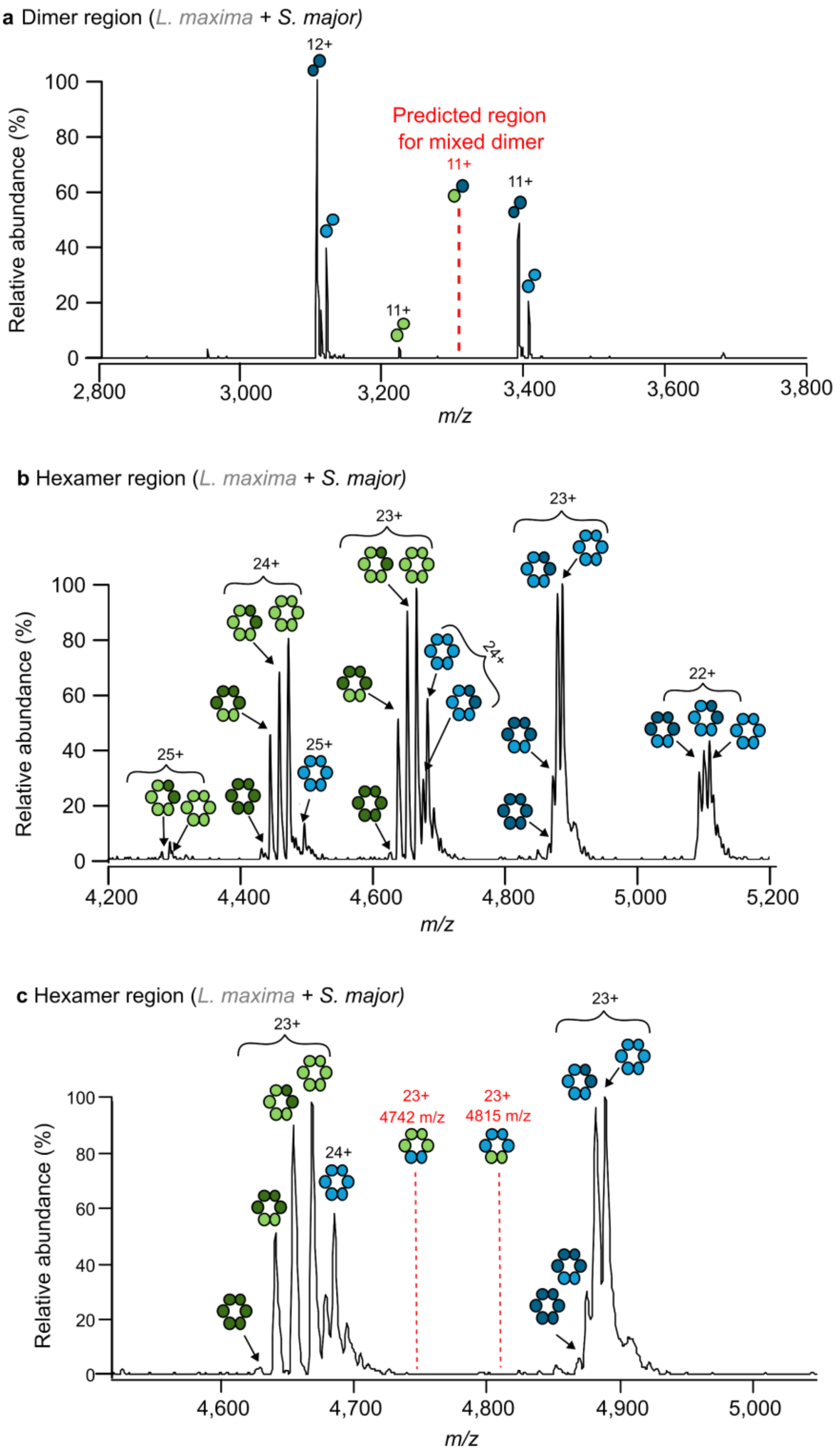

Fig S10. Native mass spectra of phycobiliprotein extract from *L. maxima* (light) mixed with *S. major* (dark). The predicted regions for mixed dimer consisting of subunits from both phycocyanin and allophycocyanin are highlighted in red (a). The mixed hexamers that were observed are shown in (b) along with the highlighted region whereby mixed phycocyanin/allophycocyanin complexes could have been observed shown in (c). The phycocyanin and allophycocyanin subunits are labelled in blue and green, respectively.

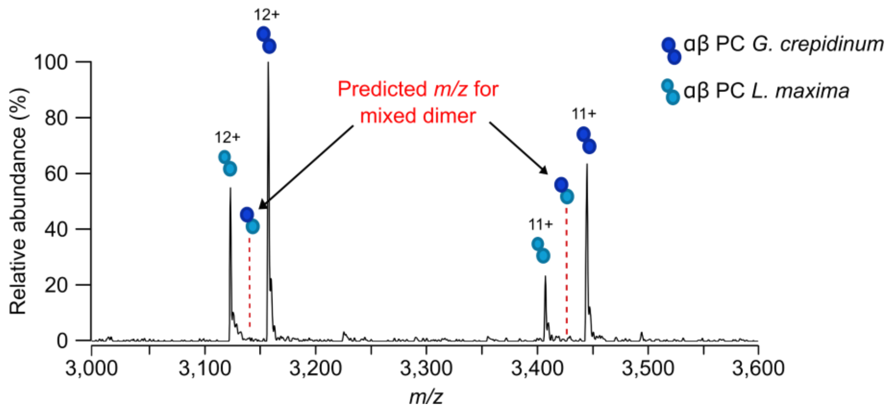

Fig S11. Native mass spectrum of mixed phycobiliprotein extracts from *L. maxima* (light) and G*. crepidinum* (dark) at low *m/z* region. No mixed dimeric phycocyanin complexes, consisting of an alpha subunit from one strain and the beta subunit of the other, were observed. The predicted regions for a mixed phycocyanin dimer are highlighted in red.

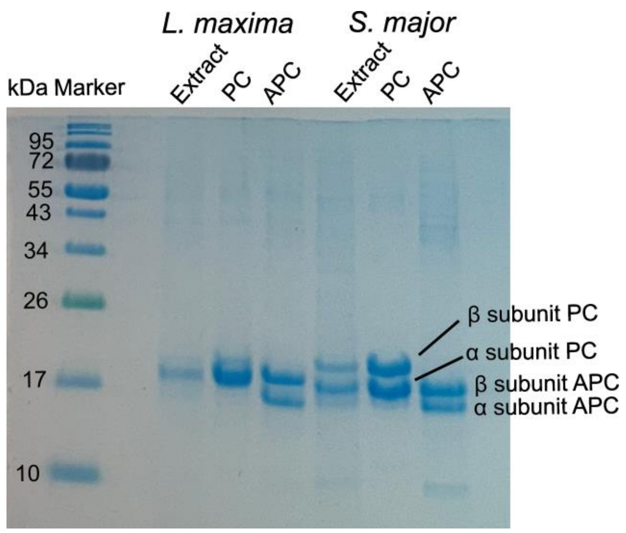

Fig S12. SDS-PAGE showing purity of phycobiliprotein extracts (extract) from *L.maxima* and *S.major*, along with the purity of phycocyanin (PC) and allophycocyanin (APC) following protein purification.

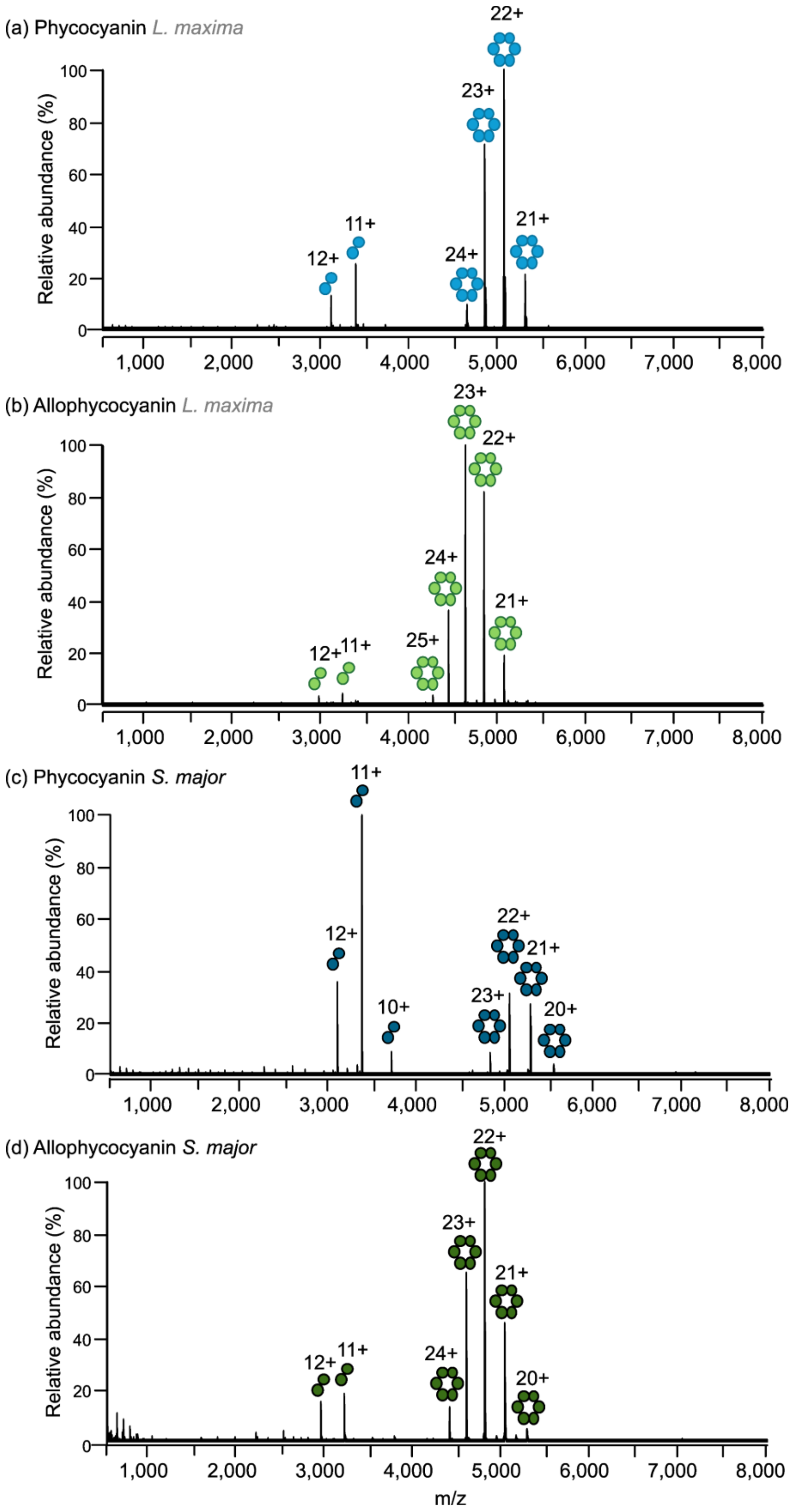

Fig S13. Native mass spectrum of purified phycocyanin (a,c) and allophyocyanin (b,d) from *L.maxima* (a,b) and *S.major* (c,d). Charge state distributions corresponding to the dimer and hexamers are shown in blue and green for phycocyanin and allophycocyanin, respectively.

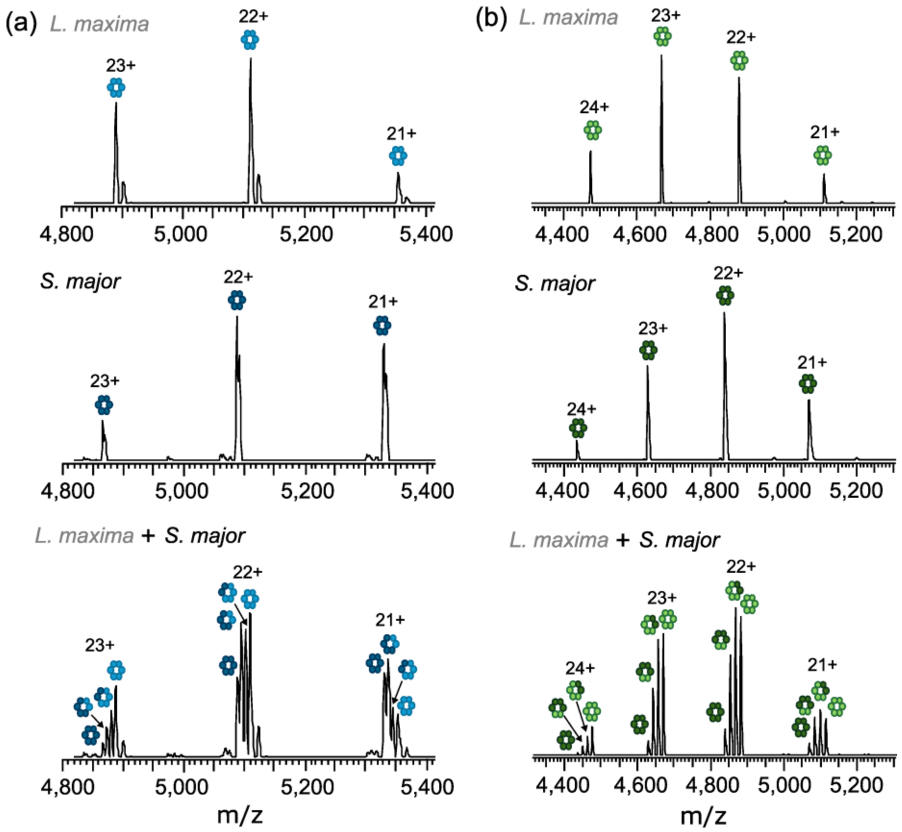

Fig S14. Native mass spectrum of mixed purified phycobiliproteins from *L. maxima* (light) and *S.major* (dark). Heterologous complex formation forms without linker protein presence with both phycocyanin (a) and allophycocyanin (b).

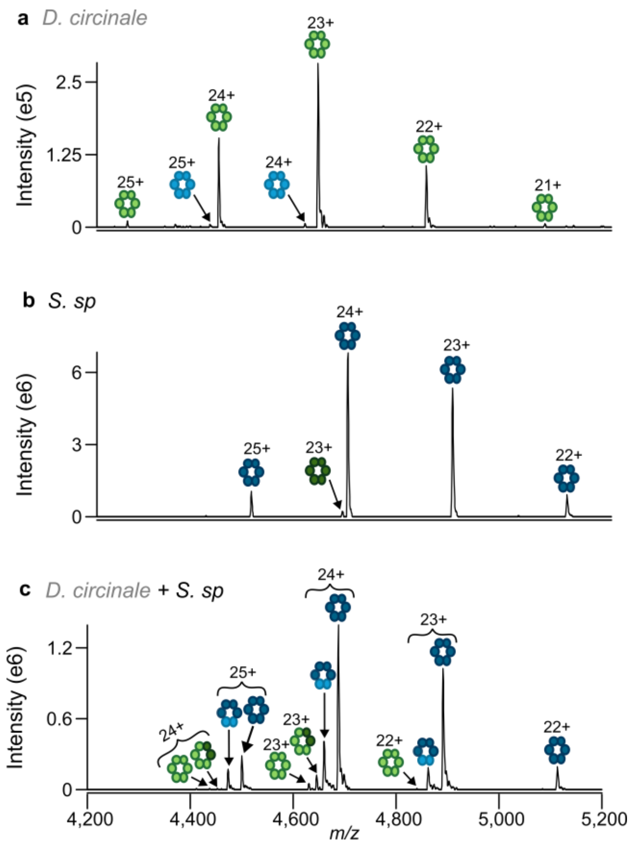

Fig S15. Native mass spectra of (αβ)₃ allophycocyanin (green) and phycocyanin (blue) complexes from *D. circinale* (a), *S. sp.* (b) alone, and from when *D. circinale* was mixed with *S. sp* (c). The αβ dimeric building blocks are colored (light or dark) according to the strains from which they originate.

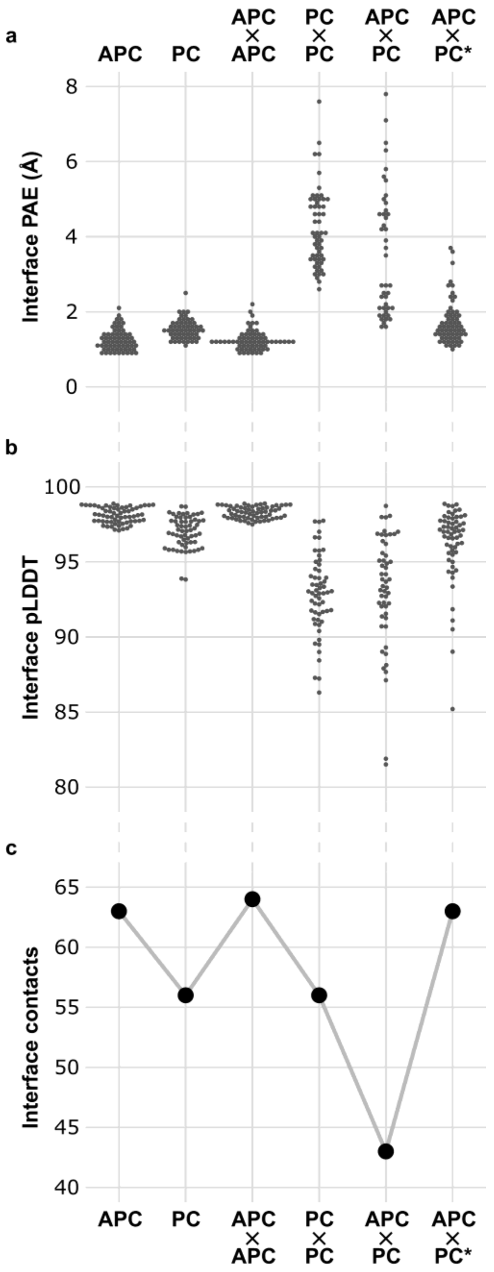

Fig S16. Statistics computed from AlphaFold2 predicted structures. APC: hexamer of *Dolichospermum circinale* CCAP 1403/21 (*D. circinale*) APC sequences. PC: hexamer of *D. circinale* PC sequences. APC x APC: hexamer containing two *D. circinale* APC dimers and one APC dimer from *Gloeomargarita lithophora* CCAP 1437/1 (*G. lithophora*). PC x PC: hexamer containing two *D. circinale* PC dimers and one *G. lithophora* PC dimer. APC x PC: hexamer containing two *D. circinale* APC dimers and one *D. circinale* PC dimer. APC x PC*: hexamer containing two *D. circinale* APC dimers and one modified *D. circinale* PC dimer. **a)** Predicted Aligned Error (PAE) for the pairs of amino acids involved in contacts in the hexamer. **b)** Predicted Local Distance Difference Test (pLDDT) for individual amino acids involved in contacts in the hexamer. **c)** Number of contacts. All statistics were computed focusing only one dimer (in structures containing more than one kind of dimers, this is the one present as a single copy).

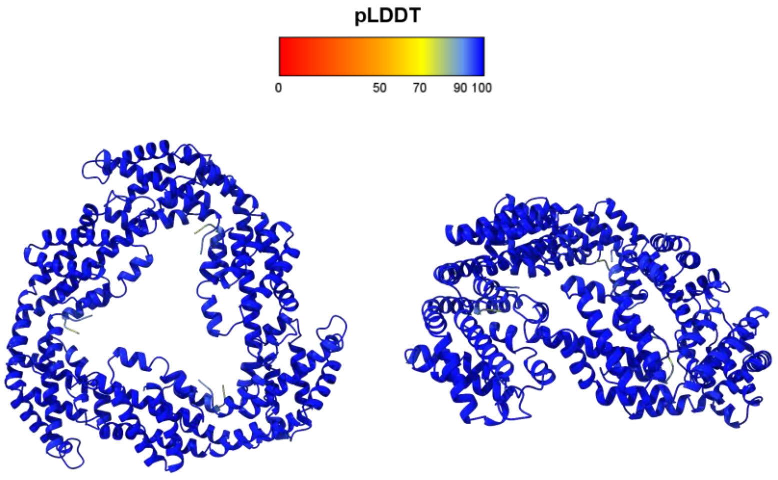

apcAAABBB_1403_21.pdb

Fig S17. Alphafold prediction of the structure of the allophycocyanin hexamer from *Dolichospermum circinale* CCAP 1403/21. Residues are coloured according to the pLDDT.

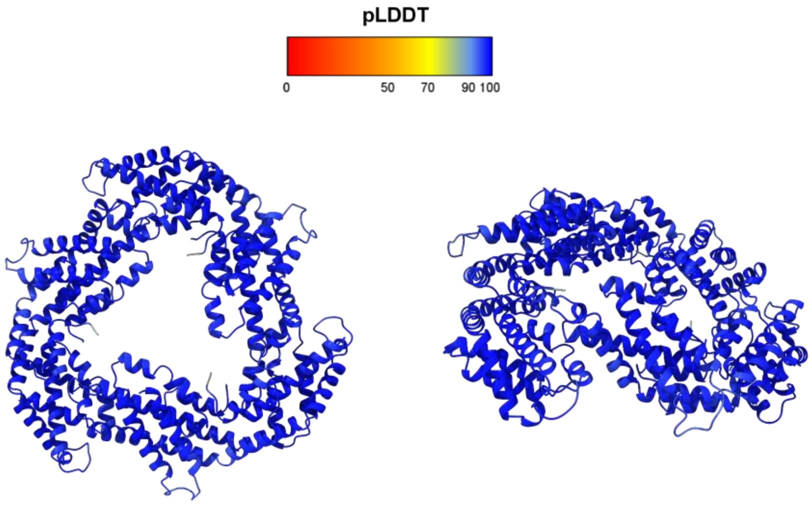

cpcAAABBB_1403_21.pdb

Fig S18. Alphafold prediction of the structure of the phycocyanin hexamer from *Dolichospermum circinale* CCAP 1403/21. Residues are coloured according to the pLDDT.

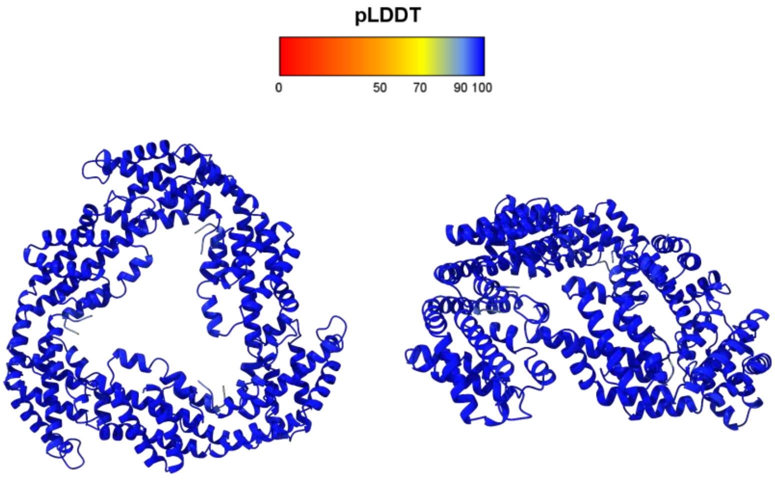

apcAABB_1403_21_apcAB_1437_1.pdb

Fig S19. Alphafold prediction of the structure of the mixed allophycocyanin hexamer containing subunits from *Dolichospermum circinale* CCAP 1403/21 and *Gloeomargarita litophora* CCAP 1437/1. Residues are coloured according to the pLDDT.

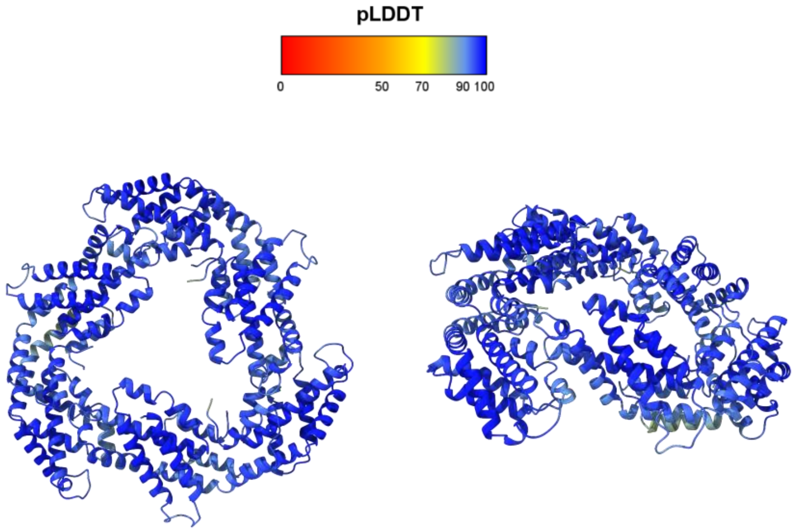

cpcAABB_1403_21_cpcAB_1437_1.pdb

Fig S20. Alphafold prediction of the structure of the mixed phycocyanin hexamer containing subunits from *Dolichospermum circinale* CCAP 1403/21 and *Gloeomargarita litophora* CCAP 1437/1. Residues are coloured according to the pLDDT.

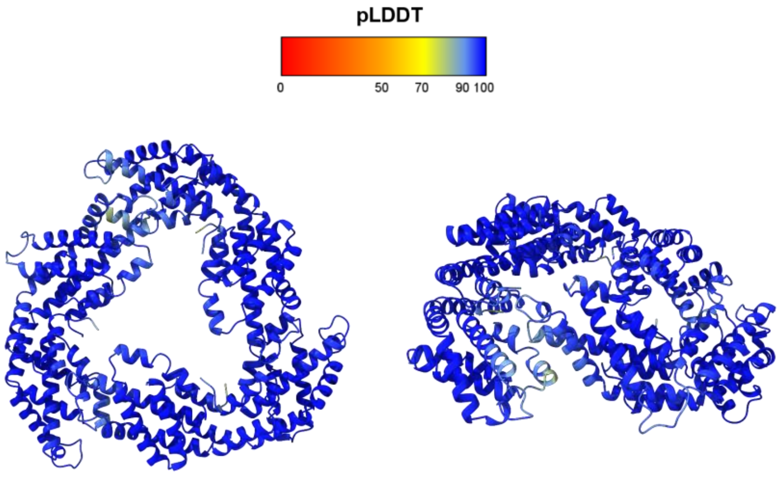

apcAABB_cpcAB_1403_21.pdb

Fig S21. Alphafold prediction of the structure of the mixed hexamer containing subunits of allophycocyanin and phycocyanin from *Dolichospermum circinale* CCAP 1403/21. Residues are coloured according to the pLDDT.

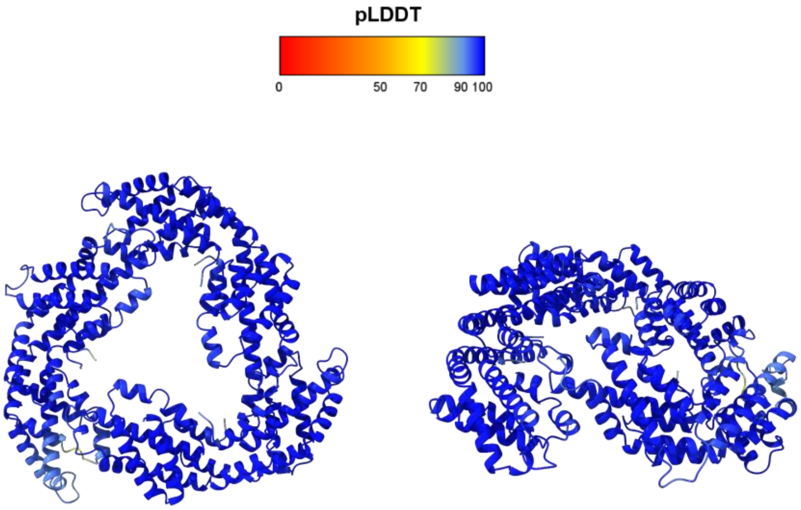

apcAABB_cpcAsBs_1403_21_2.pdb

Fig 22. Alphafold prediction of the structure of the mixed hexamer containing subunits of allophycocyanin and modified phycocyanin from *Dolichospermum circinale* CCAP 1403/21. Residues are coloured according to the pLDDT.

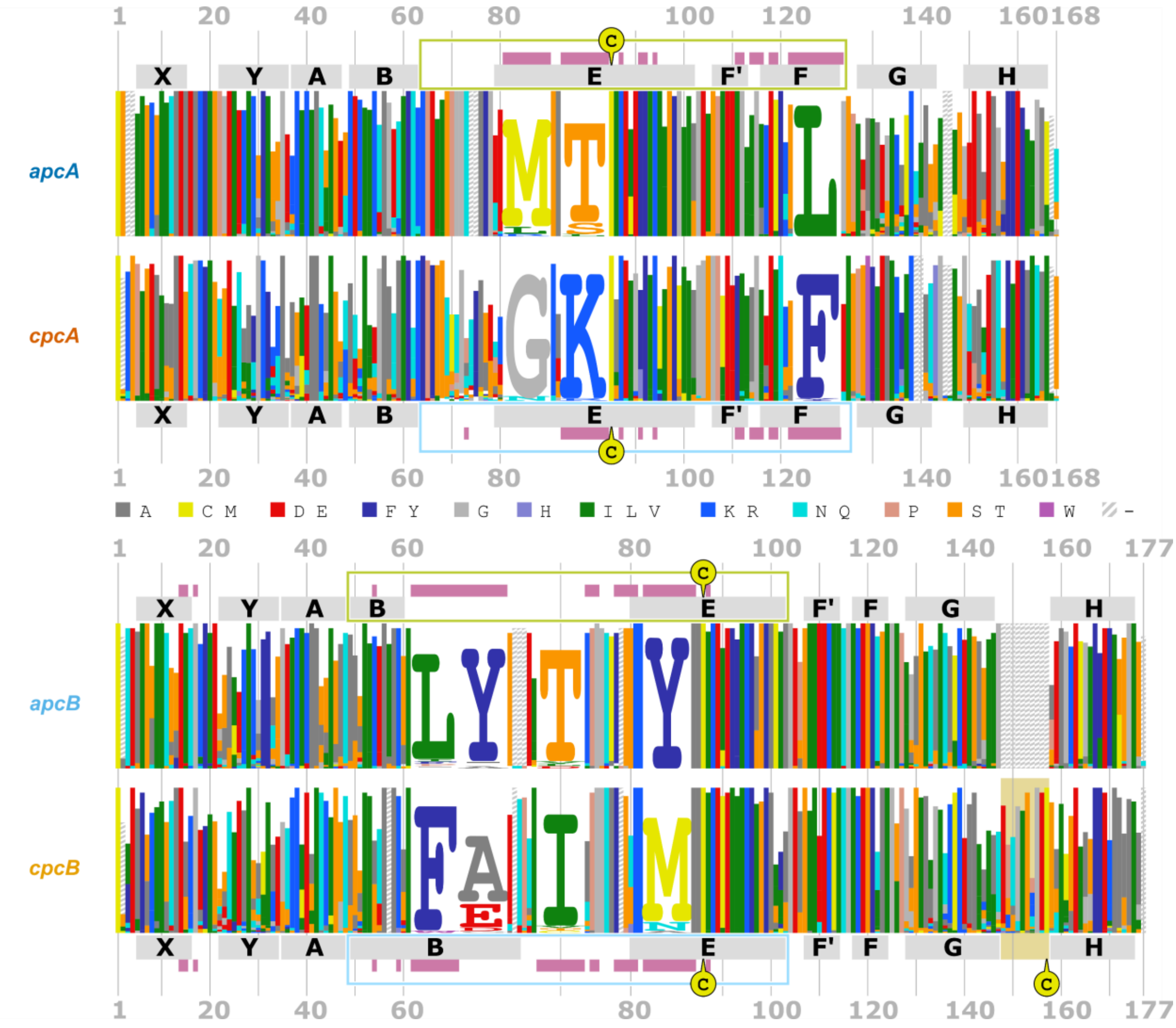

Fig S23. Comparison of APC and PC sequences. Protein-coding sequences of the genes encoding the APC and PC α and β subunits. Each column in the plot shows a color-coded representation of the proportion of sequences that have a certain amino acid in each aligned position. The total height of each column is proportional to the information content of each position. Key residues are highlighted as a sequence logo. Sequence regions corresponding to α-helices in the secondary structure of the subunit are highlighted in grey and show the name of the helix. The pink bars highlight amino acids involved in dimer-dimer contacts within hexamers. Conserved cysteine residues where phycocyanobilin is bound covalently are indicated by a “C”. Regions contoured in light blue in *cpcA* and *cpcB* were replaced with the corresponding regions highlighted in light green in *apcA* and *apcB* to produce the modified PC dimer.

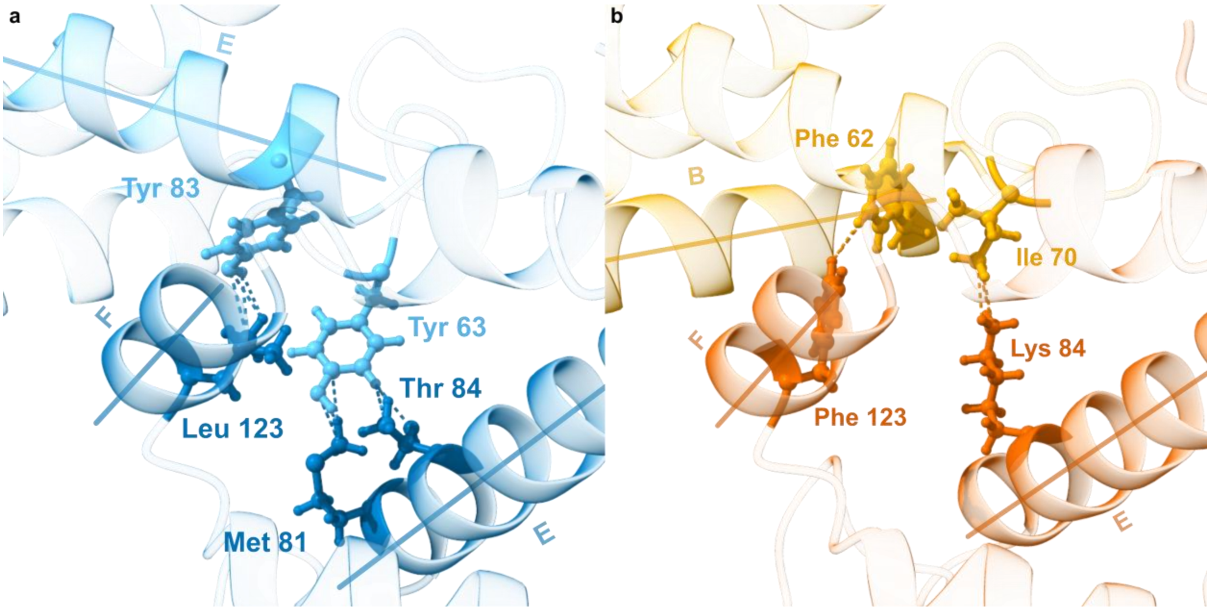

Fig S24. Comparison of APC and PC interactions. a) Selected interactions between the α and β subunits of APC in *Dolichospermum circinale* CCAP 1403/21 (*D. circinale*). Amino acid positions correspond to positions in the alignment (Figure S14). b) Selected interactions between the α and β subunits of *D. circinale* PC.

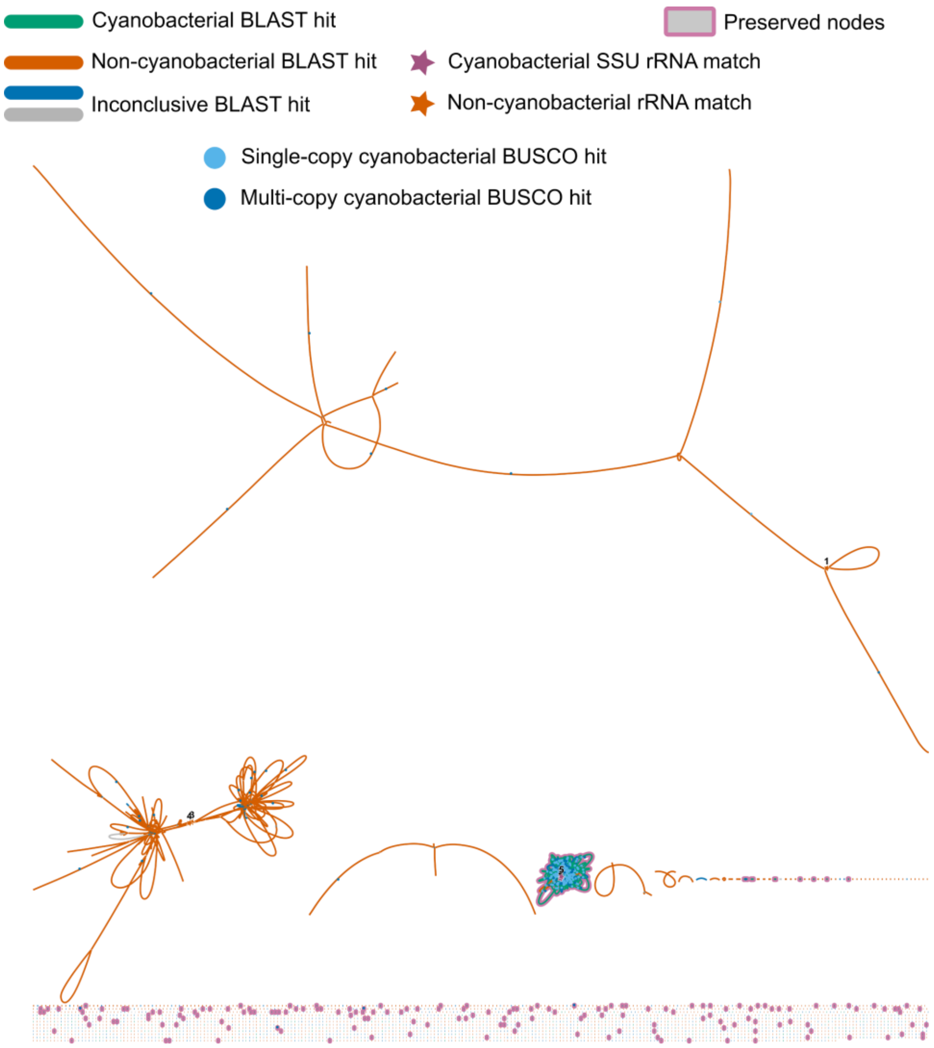

Fig S25. Bandage plot for the *Phormidesmis priestleyi* ANT.L61.2 ULC 022 genome. Nodes are color-coded based on phylogenetic assignments deriving from BLAST searches and cyanobacterial BUSCO hits are shown as dots. 16S rRNA hits are shown as stars. Nodes that were determined to represent cyanobacterial sequences and thus preserved in the final assembly are highlighted in pink.

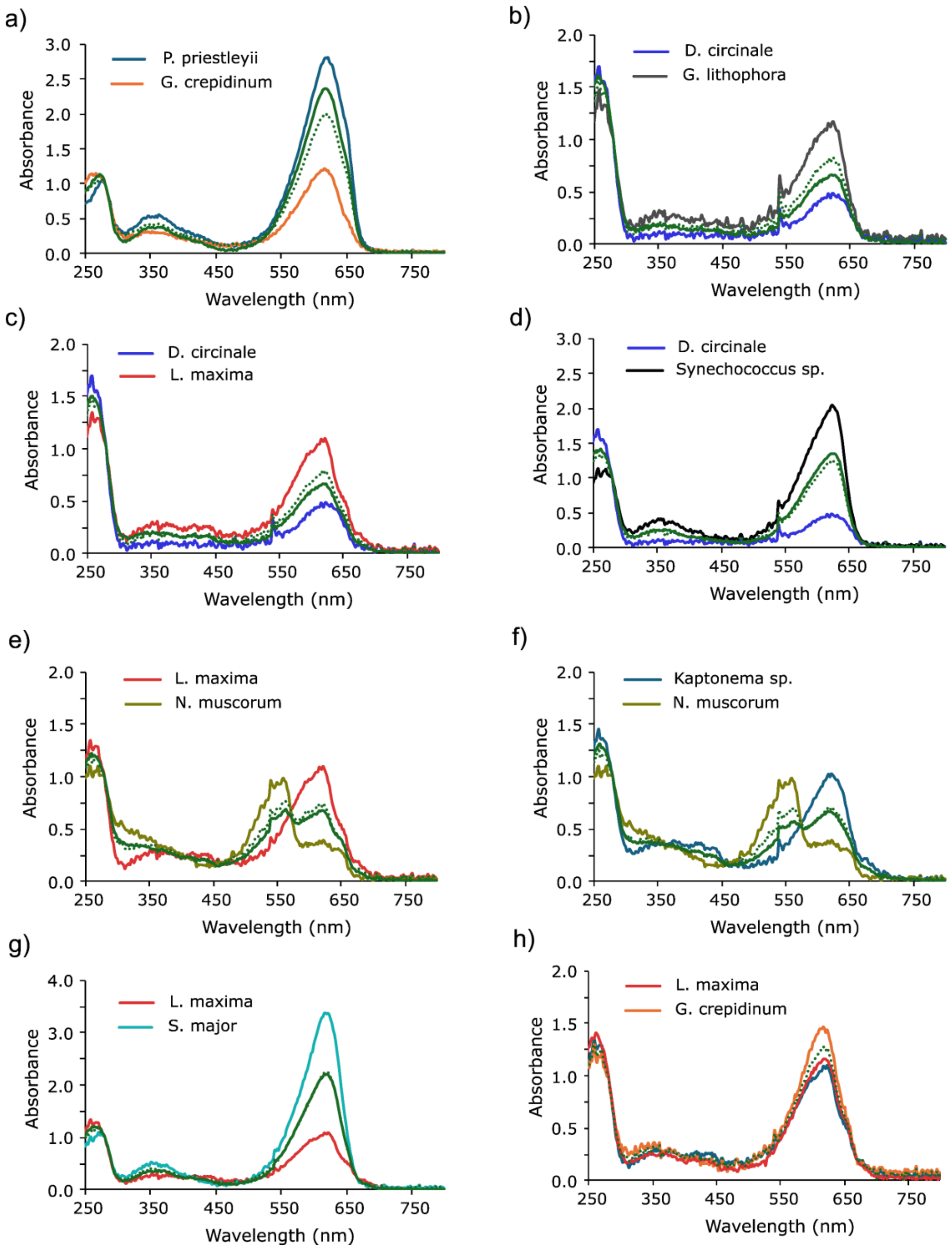

Fig S26. UV-visible spectroscopy data for phycobiliprotein extracts from all species alone and their mixtures as analyzed by native MS. The measured absorbance spectrum of the phycobiliproteins from *P. priestleyii* and *G. crepidinum* (a), *D. circinale* and *G. lithophora* (b), *D. circinale* and *L. maxima (c), D. circinale* and *Synechococcus sp.* (d), *L.maxima* and *N. muscorum* (e), *Kaptonema sp.* and *N. muscorum* (f), *L.maxima* and *S.major* (g), *L.maxima* and *G.crepidinum* (h) are shown in green. The predicted mixed absorbance spectrum based of the individual species traces are shown in each case as green dotted line. Note that *N. muscorum* expresses an additional phycobiliprotein/bilin chromophore that results in a peak at 560 nm.

Table S1. Predicted versus observed molecular weights of protein complexes detected from phycobiliprotein extracts from individual strains.

Protein Species	Predicted MW (Da)	Phycocyanobilin	N4-methylAsn	N-terminal Met loss	Observed MW (Da)	Mass Deviation (%)
Limnospira maxima						
PC (αβ)	37,468	3	1	0	37,468	<0.01
PC $(\alpha\beta)_3$	112,404	9	3	0	112,420	0.01
APC (αβ)	35,778	2	1	1	35,777	<0.01
APC $(\alpha\beta)_3$	107,335	6	3	3	107,350	0.01
APC $(\alpha\beta)_2(\alpha\text{-B}\beta)_1$	107,944	6	3	3	107,959	0.01
Gloeocapsopsis crepidinum						
PC (αβ)	37,876	3	1	0	37,878	<0.01
PC $(\alpha\beta)_3$	113,628	9	3	0	113,666	0.03
APC (αβ)	36,148	2	1	1	36,149	<0.01
APC $(\alpha\beta)_3$	108,445	6	3	3	108,478	0.03
Nostoc muscorum						
PC (αβ)	37,262	3	1	0	-	-
PC $(\alpha\beta)_3$	111,786	9	3	0	-	-
APC (αβ)	35,528	2	1	2	35,527	<0.01
APC $(\alpha\beta)_3$	106,584	6	3	6	106,601	0.02
Kamptonema sp.						
PC (αβ)	37,331	3	1	1	37,331	<0.01
PC $(\alpha\beta)_3$	111,993	9	3	3	-	-
APC (αβ)	35,821	2	1	1	-	-
APC $(\alpha\beta)_3$	107,464	6	3	3	107,475	0.01
Phormidesmis priestleyi						
PC (αβ)	37,194	3	1	0	37,195	<0.01
PC $(\alpha\beta)_3$	111,582	9	3	0	111,606	0.02
APC (αβ)	35,830	2	1	1	35,829	<0.01
APC $(\alpha\beta)_3$	107,491	6	3	3	107,509	0.02
APC $(\alpha\beta)_3$ + ApcC	115,308	6	3	3	115,269	0.03
Spirulina major						
PC (αβ)	37,307	3	1	0	37,307	<0.01
PC $(\alpha\beta)_3$	111,921	9	3	0	-	-
APC (αβ)	35,458	2	1	1	35,458	<0.01
APC $(\alpha\beta)_3$	106,375	6	3	3	106,389	0.01
Dolichospermum circinale						
PC (αβ)	36,823	3	1	1	36,823	<0.01
PC $(\alpha\beta)_3$	110,469	9	3	3	110,490	0.02
APC (αβ)	35,487	2	1	1	35,487	<0.01
APC $(\alpha\beta)_3$	106,462	6	3	3	106,477	0.01
Gloeomargarita lithophora						
PC (αβ)	37,526	3	1	0	37,526	<0.01
PC $(\alpha\beta)_3$	112,577	9	3	0	112,605	0.03

APC ($\alpha\beta$)	35,893	2	1	1	35,893	<0.01
APC ($\alpha\beta$)$_3$	107,679	6	3	3	107,698	0.02
Synechococcus sp.						
PC ($\alpha\beta$)	37,485	3	1	0	37,491	0.02
PC ($\alpha\beta$)$_3$	112,455	9	3	0	112,491	0.03
APC ($\alpha\beta$)	35,847	2	1	1	35,847	<0.01
APC ($\alpha\beta$)$_3$	107,542	6	3	3	107,559	0.02

Table S2. Predicted versus observed molecular weights of protein complexes from mixed strains.

	Phycocyanin			Allophycocyanin		
Hexamer Composition	Predicted MW (Da)	Observed MW (Da)	Mass Deviation (%)	Predicted MW (Da)	Observed MW (Da)	Mass Deviation (%)
Limnospira maxima + Gloeocapsopsis crepidinum						
$(\alpha\beta_{L.\ maxima})_3$	112,404	112,432	0.02	107,334.6	107,336.5	<0.01
$(\alpha\beta_{L.\ maxima})_2\ (\alpha\beta_{G.\ crepidinum})_1$	112,812	112,848	0.03	107,704.6	107,728.4	0.02
$(\alpha\beta_{L.\ maxima})_1\ (\alpha\beta_{G.\ crepidinum})_2$	113,220	113,271	0.04	108,074.6	108,119.3	0.04
$(\alpha\beta_{G.\ crepidinum})_3$	113,628	113,683	0.05	108,444.6	-	-
Limnospira maxima + Nostoc muscorum						
$(\alpha\beta_{L.\ maxima})_3$	112,404	112,448	0.04	107,334.6	107,370.1	0.03
$(\alpha\beta_{L.\ maxima})_2\ (\alpha\beta_{N.\ muscorum})_1$	112,198	-	-	107,084.4	107,118.5	0.03
$(\alpha\beta_{L.\ maxima})_1\ (\alpha\beta_{N.\ muscorum})_2$	111,992	-	-	106,834.2	106,873.8	0.04
$(\alpha\beta_{N.\ muscorum})_3$	111,786	-	-	106,584.0	106,606.8	0.02
Kamptonema sp. + Nostoc muscorum						
$(\alpha\beta_{Kamptonema\ sp.})_3$	111,993	-	-	107,464	107,484	0.02
$(\alpha\beta_{Kamptonema\ sp.})_2\ (\alpha\beta_{N.\ muscorum})_1$	111,924	-	-	107,179	107,184	0.01
$(\alpha\beta_{Kamptonema\ sp.})_1\ (\alpha\beta_{N.\ muscorum})_2$	111,855	-	-	106,877	106,893	0.01
$(\alpha\beta_{N.\ muscorum})_3$	111,786	-	-	106,584	106,606	0.02
Phormidesmis priestleyi + Gloeocapsopsis crepidinum						
$(\alpha\beta_{P.\ priestleyi})_3$	111,582	111,604	0.02	107,491	107,501	0.01
$(\alpha\beta_{P.\ priestleyi})_2\ (\alpha\beta_{G.\ crepidinum})_1$	112,264	-	-	107,809	107,828	0.02
$(\alpha\beta_{P.\ priestleyi})_1\ (\alpha\beta_{G.\ crepidinum})_2$	112,946	-	-	108,127	108,155	0.03
$(\alpha\beta_{G.\ crepidinum})_3$	113,628	113,655	0.02	108,445	-	-
Limnospira maxima + Spirulina major						
$(\alpha\beta_{L.\ maxima})_3$	112,404	112,428	0.02	107,335	107,359	0.02
$(\alpha\beta_{L.\ maxima})_2\ (\alpha\beta_{S.\ major})_1$	112,243	112,257	0.01	107,015	107,045	0.03
$(\alpha\beta_{L.\ maxima})_1\ (\alpha\beta_{S.\ major})_2$	112,082	112,095	0.01	106,695	106,714	0.02
$(\alpha\beta_{S.\ major})_3$	111,921	111,937	0.01	106,375	106,394	0.02
Dolichospermum circinale + Limnospira maxima						
$(\alpha\beta_{D.\ circinale})_3$	110,469	-	-	106,462	-	-
$(\alpha\beta_{D.\ circinale})_2\ (\alpha\beta_{L.\ maxima})_1$	111,114	-	-	106,753	106,775	0.02
$(\alpha\beta_{D.\ circinale})_1\ (\alpha\beta_{L.\ maxima})_2$	111,759	-	-	107,044	107,072	0.03
$(\alpha\beta_{L.\ maxima})_3$	112,404	112,431	0.02	107,335	107,359	0.02

Dolichospermum circinale + Gloeomargarita lithophora						
$(\alpha\beta_{D.\ circinale})_3$	110,469	-	-	106,462	106,501	0.04
$(\alpha\beta_{D.\ circinale})_2\ (\alpha\beta_{G.\ lithophora})_1$	111,171	-	-	106,867	106,897	0.03
$(\alpha\beta_{D.\ circinale})_1\ (\alpha\beta_{G.\ lithophora})_2$	111,874	111,926	0.05	107,273	107,288	0.01
$(\alpha\beta_{G.\ lithophora})_3$	112,577	112,645	0.06	107,679	-	-
Dolichospermum circinale + Synechococcus sp.						
$(\alpha\beta_{D.\ circinale})_3$	110,469	-	-	106,462	106,486	0.02
$(\alpha\beta_{D.\ circinale})_2\ (\alpha\beta_{S.\ sp.})_1$	111,131	-	-	106,822	106,850	0.03
$(\alpha\beta_{D.\ circinale})_1\ (\alpha\beta_{S.\ sp.})_2$	111,793	111,829	0.03	107,182	-	-
$(\alpha\beta_{S.\ sp.})_3$	112,455	112,494	0.03	107,542	-	-

Table S3. Summary of native MS data of mixed phycobiliprotein extracts. The predicted mixed $(\alpha\beta)_3$ phycocyanin (PC) and allophycocyanin (APC) complexes are reported along with the complexes detected by native MS. Note that PC and APC were not detected simultaneously for all cyanobacterial strains preventing observation of subunit-exchanged complexes when these strains were mixed.

			Mixtures							
		Strain 1	*L. maxima*	*L. maxima*	*Kamptonema sp.*	*P. priestleyi*	*L. maxima*	*D. circinale*	*D. circinale*	*D. circinale*
		Strain 2	*G. crepidinum*	*N. muscorum*	*N. muscorum*	*G. crepidinum*	*S. major*	*L. maxima*	*G. lithophora*	*S. sp.*
Strain 1 Observed Complex	PC	$\alpha\beta$	X	X	X	X	X	X	X	X
		$(\alpha\beta)_3$	X	X		X	X	X	X	X
	APC	$\alpha\beta$	X	X		X	X	X	X	X
		$(\alpha\beta)_3$	X	X	X	X	X	X	X	X
Strain 2 Observed Complex	PC	$\alpha\beta$	X			X	X	X	X	X
		$(\alpha\beta)_3$	X			X		X	X	X
	APC	$\alpha\beta$	X	X	X	X	X	X	X	X
		$(\alpha\beta)_3$	X	X	X	X	X	X	X	X
Predicted Complexes in Mixture	PC	$(\alpha\beta_1)_3$	X	X		X	X	X	X	X
		$(\alpha\beta_1)_2(\alpha\beta_2)_1$	X			X		X	X	X
		$(\alpha\beta_1)_1(\alpha\beta_2)_2$	X			X		X	X	X
		$(\alpha\beta_2)_3$	X			X		X	X	X
	APC	$(\alpha\beta_1)_3$	X	X	X	X	X	X	X	X
		$(\alpha\beta_1)_2(\alpha\beta_2)_1$	X	X	X	X	X	X	X	X
		$(\alpha\beta_1)_1(\alpha\beta_2)_2$	X	X	X	X	X	X	X	X
		$(\alpha\beta_2)_3$	X	X	X	X	X	X	X	X
Observed Complexes in Mixture	PC	$(\alpha\beta_1)_3$	X	X		X	X			
		$(\alpha\beta_1)_2(\alpha\beta_2)_1$	X				X			
		$(\alpha\beta_1)_1(\alpha\beta_2)_2$	X				X		X	X
		$(\alpha\beta_2)_3$	X			X		X	X	X
	APC	$(\alpha\beta_1)_3$	X	X	X	X	X		X	X
		$(\alpha\beta_1)_2(\alpha\beta_2)_1$	X	X	X	X	X	X	X	X
		$(\alpha\beta_1)_1(\alpha\beta_2)_2$	X	X	X	X	X	X	X	
		$(\alpha\beta_2)_3$		X	X		X	X		

Table S4. RMSD between AlphaFold2 predicted structures. *D. circinale*: *Dolichospermum circinale* CCAP 1403/21; *G. lithophora*: *Gloeomargarita lithophora* CCAP 1437/1. For the *D. circinale* APC vs *D. circinale* PC comparison, the table shows the RMSD between aligned amino acid pairs that are closer than 2Å, as well as between all aligned amino acid pairs (in brackets). For all other comparisons, the two values are identical.

Comparison			RMSD (Å)
D. circinale APC	vs	*G. lithophora* APC	0.171
D. circinale PC	vs	*G. lithophora* PC	0.389
D. circinale APC	vs	*G. lithophora* PC	0.947 (1.853)
D. circinale *apcA*	vs	*G. lithophora apcA*	0.199
D. circinale *apcB*	vs	*G. lithophora apcB*	0.131
D. circinale *cpcA*	vs	*G. lithophora cpcA*	0.360
D. circinale *cpcB*	vs	*G. lithophora cpcB*	0.259

Table S5. Cyanobacterial genomes used for the phylogenomic analysis. Strains that were experimentally analyzed in this study are highlighted in bold.

Strain name	Genome assembly accession
**Dolichospermum circinale CCAP 1403/21**	**JBHYCU000000000**
**Gloeocapsopsis crepidinum CCAP 1425/1**	**JBHYDD000000000**
**Gloeomargarita lithophora CCAP 1437/1**	**JBHYDV000000000**
**Kamptonema sp. SAMS 01UC**	**JBHYGB000000000**
**Limnospira maxima CCAP 1475/9**	**JBIMLI000000000**
**Nostoc muscorum CCAP 1453/12**	**JBHYEF000000000**
**Phormidesmis priestleyi ANT.L61.2**	**JBHLFI000000000**
**Spirulina major CCAP 1475/3**	**JBIMLG000000000**
Acaryochloris marina MBIC11017	GCF_000018105.1_ASM1810v1
Anabaena cylindrica PCC 7122	GCF_002367955.1_ASM236795v1
Anthocerotibacter panamensis C109	GCF_018389385.1_ASM1838938v1
Ca. Synechococcus spongiarum SH4	GCF_000586015.1_SynSpo.0
Calothrix sp. PCC 6303	GCF_000317435.1_ASM31743v1
Chamaesiphon minutus PCC 6605	GCF_000317145.1_ASM31714v1
Chlorogloeopsis fritschii PCC 6912	GCF_003990575.1_ASM399057v1
Chroococcidiopsis thermalis PCC 7203	GCF_000317125.1_ASM31712v1
Coleofasciculus chthonoplastes PCC 7420	GCF_000155555.1_ASM15555v1
Crinalium epipsammum PCC 9333	GCF_000317495.1_ASM31749v1
Cyanobacterium stanieri PCC 7202	GCF_000317655.1_ASM31765v1
Desertifilum tharense IPPAS B-1220	GCF_001746915.1_ASM174691v1
Gloeobacter violaceus PCC 7421	GCF_000011385.1_ASM1138v1
Gloeocapsa sp. PCC 7428	GCF_000317555.1_ASM31755v1
Kamptonema formosum PCC 6407	GCF_000332155.1_ASM33215v1
Leptolyngbya ohadii IS1	GCF_002215035.1_ASM221503v1
Leptolyngbya sp. CCY15150	GCF_016888135.1_ASM1688813v1
Leptolyngbya sp. 'hensonii'	GCF_001939115.1_ASM193911v1
Leptolyngbya sp. PCC 7375	GCF_000316115.1_ASM31611v1
Lusitaniella coriacea LEGE 07157	GCF_015207425.1_ASM1520742v1
Microcoleus sp. FACHB-831	GCF_014695585.1_ASM1469558v1
Moorena producens 3L	GCF_000211815.1_ASM21181v1
Nodosilinea nodulosa PCC 7104	GCF_000309385.1_ASM30938v1
Nodularia spumigena CCY9414	GCF_000340565.2_ASM34056v3
Nostoc punctiforme PCC 73102	GCF_000020025.1_ASM2002v1
Oscillatoria sp. PCC 10802	GCF_000332335.1_ASM33233v1
Phormidesmis priestleyi BC1401	GCF_001650195.1_ASM165019v1
Pleurocapsa sp. PCC 7319	GCF_000332195.1_ASM33219v1
Prochlorococcus marinus str. MIT 9313	GCF_000011485.1_ASM1148v1
Prochloron didemni P2-Fiji	GCF_000252425.1_ASM25242v1
Prochlorothrix hollandica PCC 9006 = CALU 1027	GCF_000332315.1_ASM33231v1
Pseudanabaena sp. BC1403	GCF_002914585.1_ASM291458v1
Pseudanabaena sp. PCC 7367	GCF_000317065.1_ASM31706v1
Roseofilum reptotaenium AO1-A	GCA_001890975.1_ASM189097v1

Scytonema hofmannii PCC 7110	GCF_000346485.2_ASM34648v2
Spirulina subsalsa PCC 9445	GCF_000314005.1_ASM31400v1
Synechococcus elongatus PCC 6301	GCF_022984195.1_ASM2298419v1
Synechococcus sp. C9	GCF_022984075.1_ASM2298407v1
**Synechococcus sp. CCAP 1479/10**	**GCF_019038515.1_ASM1903851v1**
Synechococcus sp. JA-3-3Ab	GCF_000013205.1_ASM1320v1
Synechococcus sp. PCC 7336	GCF_000332275.1_ASM33227v1
Synechocystis sp. PCC 6803	GCF_011392055.1_ASM1139205v1
Thermostichus vulcanus str. 'Rupite'	GCF_022848905.1_ASM2284890v1
Thermosynechococcus vestitus BP-1	GCF_000011345.1_ASM1134v1
Tolypothrix sp. NIES-4075	GCF_002218085.1_ASM221808v1
Tolypothrix sp. PCC 7910	GCF_011769525.1_ASM1176952v1
Vulcanococcus limneticus LL	GCF_002252705.1_ASM225270v1

Table S6. BLAST query sequences for APC and PC subunits.

Gene	Query sequence accessions
apcA	WP_218080831.1, WP_019501514.1, WP_017326497.1, WP_235278780.1, NJM98305.1, WP_042156917.1, WP_197156845.1, WP_204103584.1, WP_194028217.1, WP_167726827.1
apcB	WP_080813475.1, O68970.1, ELS01491.1, ELS02790.1, P50031.1, EDX87068.1, P06113.1, Q01952.1, AFY30991.1, WP_096678231.1
cpcA	P00307.3, P00308.3, P13530.2, P03943.1, P07121.3, P50032.1, P72509.2, Q54715.1, P20776.2
cpcB	P00310.3, P00312.3, P06539.2, P72508.2, Q54714.2, P03944.1, P07120.4, P50033.1, P20777.1

Table S7. Outgroup sequences for APC and PC subunits.

Outgroup for	Gene	Accessions
apcA	***apcA2***	WP_015191756.1, MBE9044050.1, WP_015196848.1, WP_127084554.1, WP_017289058.1, WP_190798666.1, WP_035984340.1, WP_017298278.1, WP_015188832.1, WP_096563448.1
	apcD1	BAY50952.1, P11390.3, WP_096626391.1, WP_096660407.1, EDX84316.1, WP_080807085.1, WP_073601446.1, P72870.1, O68966.1, WP_106242755.1
	apcD2	EDX85573.1, WP_080806387.1, WP_073600232.1, WP_106235913.1, OWY64182.1, AFY30760.1, WP_096658609.1, WP_096620788.1, WP_009453700.1, WP_016873422.1
	apcD3	AFY86568.1, EDX86739.1, WP_080806384.1, WP_106235919.1, WP_073600234.1, WP_096658611.1, WP_016873424.1, WP_096620786.1, NJR61197.1, WP_015126586.1
	apcD4	WP_080809693.1, WP_006454943.1, ELS00905.1, WP_106232622.1, WP_016874155.1, WP_026735770.1, AFZ32298.1, OWY64457.1, PSB46407.1, PSM45859.1
	apcD5	WP_006456289.1, WP_015153113.1, WP_096620790.1, WP_015126590.1, WP_223046610.1, WP_106235909.1, RNJ67176.1, WP_080806390.1, WP_096680233.1, MBE7379955.1
	apcF	GGA18138.1, AAB87964.1, GDX75033.1, AVH76592.1, APB34571.1, BBC25000.1, VEP13518.1, AAC14717.1
	ApcF2	WP_015153831.1, WP_192154186.1, NJM88006.1, NJP18393.1, MCL6433471.1, WP_223046947.1, WP_224344134.1, NJP09556.1, WP_009769098.1, MBW4554243.1
apcB	***apcB2***	WP_006454442.1, WP_080806389.1, WP_073600231.1, WP_106235911.1, OWY64181.1, WP_096658608.1, AFY30761.1, WP_096620789.1, WP_026734725.1, WP_096680235.1
	apcB3	WP_193930287.1, WP_026735771.1, WP_071455396.1, WP_169616007.1, WP_194029372.1, WP_006512042.1, WP_193995479.1, OWY64454.1, WP_080809690.1, WP_006453385.1

Table S8. Sequences used for AlphaFold2 structure prediction. *D. circinale*: *Dolichospermum circinale* CCAP 1403/21; *G. lithophora*: *Gloeomargarita lithophora* CCAP 1437/1. The modified *D. circinale* CpcA and CpcB sequences were obtained by combining parts of the *D. circinale* CpcA and CpcB sequences (*italics*) with parts of ApcA and ApcB from the same strain (**bold**, highlighted in **Fig S23**).

Name	Sequence
D. circinale cpcA	*MKTPITEAIASADTQGRFLSNTELQAVNGRLVRAAASMEAARGLTANAQKLIDGATSAVYSK*FPYTTSTQGNQFAADPRGKAKCARDVGHYLRIITYSLVAGGTGPLDEFLIAGLAEVNAAFDL*SPSWYVEALKSIKASHGLSGQAANEANTYIDYAINALS*
D. circinale cpcB	*MTLDVFSKVVSQADARGEFLSTEQLDALSAVVASGNKRLDAVNRITSNA*SAIVTNAARSLFEEQPQLIAPGGNAYTNRRNAACLRDMEIILRYVTYAAIAGDA*SVLDDRCLNGLRETYQALGTPGSSVAVGVGKMKEAAIAIVNDPNGITKGDCSSLVSELASYFDRAAAAVV*
D. circinale apcA	MSIVTKAIVNADAEARYLSPGELDRIKGFVAGGAQRLRIAQVLTENRERIVKQAGDQLFQK**RPDVVSPGGNAYGQEMTATCLRDLDYYLRLVTYGIVSGDVTAIEEIGIVGVREMYKSLG**TPIDAVAGGVAAMKNVAATLLSAEDSGEAGSYFDYVVGAMQ
D. circinale apcB	MQDAITSVINSSDVQGKYLDTAALEKLKGYFATGELRVRAATTISANA**AAIVKEAVAKSLLYSDITRPGGNMYTTRRYAACIRDLDYYLRYSTYAMLAGDA**SILDERVLNGLKETYNSLGVPVGATIQAISAMKEVTASLVGPDAGKEMGVYFDYISSGLS
G. lithophora cpcA	MSIVTKSIVNADAEARYLSPGELDRIKSFVSSGETRLRIAQVLSNNRERIVKEAGQQLFQKRPDVVSPGGNAYGEEMTATCLRDLDYYLRLVTYGIVSGDVTPIEEIGLVGVKEMYNSLGTPIPAVAEGVRAMKGAAGSLMSGEDATEAGFYFDFIVSAMQ
G. lithophora cpcB	MQDAITAVINSADVQGKYLDSSALDRLKKYFQTGELRVRAAATIAANAATIIKEAVAKSLLYSDITRPGGNMYTTRRYAACIRDLDYYLRYATYAMLAGDASILDERVLNGLKETYNSLGVPIGATIQSIQSMKDVTAGLVGPDAGKEMAVYFDYICSGLS
G. lithophora apcA	MKTPLTEIIASADSEGRFLSNNELQSAFGRFGKAQAGLQAAKELTAKSDQLINQAAQAVYSKFPYTTQMQGNEYASDERGKAKCARDIGYYLRMVTYCLIVGGTGPMDEYLVAGLDEINSSFNLSPSWYVEALKSIKANHGLSGDSSVQANGFIDYAINALS
G. lithophora apcB	MLDAFAKVVAQADTRGEFISTSQIDALSAMVAESNKRMDSVNRLTSNAAAIVTNAARSLFAEQPQLIQPGGNAYTNRRMAACLRDMEIILRYVTYAVLAGDASVLDDRCLNGLRETYQALGVPGASVAVGVQKMKEAAVSIVNDPTGITKGDCSQLVSEIASYFDRAAAAVV
**Modified *D. circinale cpcA***	*MKTPITEAIASADTQGRFLSNTELQAVNGRLVRAAASMEAARGLTANAQKLIDGATSAVYSK***RPDVVSPGGNAYGQEMTATCLRDLDYYLRLVTYGIVSGDVTAIEEIGIVGVREMYKSLG***SPSWYVEALKSIKASHGLSGQAANEANTYIDYAINALS*
**Modified *D. circinale cpcB***	*MTLDVFSKVVSQADARGEFLSTEQLDALSAVVASGNKRLDAVNRITSNA***AAIVKEAVAKSLLYSDITRPGGNMYTTRRYAACIRDLDYYLRYSTYAMLAGDA***SVLDDRCLNGLRETYQALGTPGSSVAVGVGKMKEAAIAIVNDPNGITKGDCSSLVSELASYFDRAAAAVV*

References

1. Synechococcus sp. CCAP 1479/10 genome assembly ASM1903851v1. *NCBI*. Available at: https://www.ncbi.nlm.nih.gov/datasets/genome/GCF_019038515.1/ [Accessed 30 October 2024].

2. M. Martin, Cutadapt removes adapter sequences from high-throughput sequencing reads. *EMBnet.journal* **17**, 10–12 (2011).

3. Joshi, N.A., Fass, J.N., Sickle: A sliding-window, adaptive, quality-based trimming tool for FastQ files (Version 1.33) [Software]. Available at https://github.com/najoshi/sickle. (2011).

4. G. V. Uritskiy, J. DiRuggiero, J. Taylor, MetaWRAP—a flexible pipeline for genome-resolved metagenomic data analysis. *Microbiome* **6**, 158 (2018).

5. Babraham Bioinformatics - FastQC A Quality Control tool for High Throughput Sequence Data. Available at: https://www.bioinformatics.babraham.ac.uk/projects/fastqc/ [Accessed 28 March 2024].

6. J. S. Boden, M. Grego, H. Bolhuis, P. Sánchez-Baracaldo, Draft genome sequences of three filamentous cyanobacteria isolated from brackish habitats. *J. Genomics* **9**, 20–25 (2021).

7. N. A. M. Chrismas, G. Barker, A. M. Anesio, P. Sánchez-Baracaldo, Genomic mechanisms for cold tolerance and production of exopolysaccharides in the Arctic cyanobacterium Phormidesmis priestleyi BC1401. *BMC Genomics* **17**, 533 (2016).

8. N. A. M. Chrismas, C. J. Williamson, M. L. Yallop, A. M. Anesio, P. Sánchez-Baracaldo, Photoecology of the Antarctic cyanobacterium Leptolyngbya sp. BC1307 brought to light through community analysis, comparative genomics and in vitro photophysiology. *Mol. Ecol.* **27**, 5279–5293 (2018).

9. A. Prjibelski, D. Antipov, D. Meleshko, A. Lapidus, A. Korobeynikov, Using SPAdes De Novo Assembler. *Curr. Protoc. Bioinforma.* **70**, e102 (2020).

10. A. Mikheenko, A. Prjibelski, V. Saveliev, D. Antipov, A. Gurevich, Versatile genome assembly evaluation with QUAST-LG. *Bioinformatics* **34**, i142–i150 (2018).

11. R. R. Wick, M. B. Schultz, J. Zobel, K. E. Holt, Bandage: interactive visualization of de novo genome assemblies. *Bioinformatics* **31**, 3350–3352 (2015).

12. M. Manni, M. R. Berkeley, M. Seppey, F. A. Simão, E. M. Zdobnov, BUSCO Update: Novel and Streamlined Workflows along with Broader and Deeper Phylogenetic Coverage for Scoring of Eukaryotic, Prokaryotic, and Viral Genomes. *Mol. Biol. Evol.* **38**, 4647–4654 (2021).

13. K. Lagesen, *et al.*, RNAmmer: consistent and rapid annotation of ribosomal RNA genes. *Nucleic Acids Res.* **35**, 3100–3108 (2007).

14. C. Camacho, *et al.*, BLAST+: architecture and applications. *BMC Bioinformatics* **10**, 421 (2009).

15. Home - Nucleotide - NCBI. Available at: https://www.ncbi.nlm.nih.gov/nucleotide/ [Accessed 28 March 2024].

16. BLAST: Basic Local Alignment Search Tool. Available at: https://blast.ncbi.nlm.nih.gov/Blast.cgi [Accessed 28 March 2024].

17. J. Alneberg, *et al.*, Binning metagenomic contigs by coverage and composition. *Nat. Methods* **11**, 1144–1146 (2014).

18. Y.-W. Wu, B. A. Simmons, S. W. Singer, MaxBin 2.0: an automated binning algorithm to recover genomes from multiple metagenomic datasets. *Bioinforma. Oxf. Engl.* **32**, 605–607 (2016).

19. K. Dd, *et al.*, MetaBAT 2: an adaptive binning algorithm for robust and efficient genome reconstruction from metagenome assemblies. *PeerJ* **7** (2019).

20. D. H. Parks, M. Imelfort, C. T. Skennerton, P. Hugenholtz, G. W. Tyson, CheckM: assessing the quality of microbial genomes recovered from isolates, single cells, and metagenomes. *Genome Res.* **25**, 1043–1055 (2015).

21. P.-A. Chaumeil, A. J. Mussig, P. Hugenholtz, D. H. Parks, GTDB-Tk v2: memory friendly classification with the genome taxonomy database. *Bioinforma. Oxf. Engl.* **38**, 5315–5316 (2022).

22. T. Seemann, Prokka: rapid prokaryotic genome annotation. *Bioinforma. Oxf. Engl.* **30**, 2068–2069 (2014).

23. M. Boetzer, C. V. Henkel, H. J. Jansen, D. Butler, W. Pirovano, Scaffolding pre-assembled contigs using SSPACE. *Bioinformatics* **27**, 578–579 (2011).

24. B. Langmead, S. L. Salzberg, Fast gapped-read alignment with Bowtie 2. *Nat. Methods* **9**, 357–359 (2012).

25. P. Danecek, *et al.*, Twelve years of SAMtools and BCFtools. *GigaScience* **10**, giab008 (2021).

26. V. M. Markowitz, *et al.*, IMG: the integrated microbial genomes database and comparative analysis system. *Nucleic Acids Res.* **40**, D115–D122 (2012).
