# Supplementary material for: Mass spectrometry reveals the evolutionary conservation of phycobiliprotein complexes
Source: Nat Commun. 2026 Feb 16;17:2834. doi: 10.1038/s41467-026-69558-y (PMC13022049; doi:10.1038/s41467-026-69558-y)
Supplement: Supplementary file 2 — Reporting Summary [file 41467_2026_69558_MOESM2_ESM.pdf]

nature portfolio

Corresponding author(s): Aneika Leney

Last updated by author(s): Jun 13, 2025

Reporting Summary

Nature Portfolio wishes to improve the reproducibility of the work that we publish. This form provides structure for consistency and transparency in reporting. For further information on Nature Portfolio policies, see our Editorial Policies and the Editorial Policy Checklist.

Statistics

For all statistical analyses, confirm that the following items are present in the figure legend, table legend, main text, or Methods section.

n/a	Confirmed	
☐	☒	The exact sample size (*n*) for each experimental group/condition, given as a discrete number and unit of measurement
☒	☐	A statement on whether measurements were taken from distinct samples or whether the same sample was measured repeatedly
☒	☐	The statistical test(s) used AND whether they are one- or two-sided<br>*Only common tests should be described solely by name; describe more complex techniques in the Methods section.*
☒	☐	A description of all covariates tested
☒	☐	A description of any assumptions or corrections, such as tests of normality and adjustment for multiple comparisons
☒	☐	A full description of the statistical parameters including central tendency (e.g. means) or other basic estimates (e.g. regression coefficient) AND variation (e.g. standard deviation) or associated estimates of uncertainty (e.g. confidence intervals)
☒	☐	For null hypothesis testing, the test statistic (e.g. *F*, *t*, *r*) with confidence intervals, effect sizes, degrees of freedom and *P* value noted<br>*Give P values as exact values whenever suitable.*
☒	☐	For Bayesian analysis, information on the choice of priors and Markov chain Monte Carlo settings
☒	☐	For hierarchical and complex designs, identification of the appropriate level for tests and full reporting of outcomes
☒	☐	Estimates of effect sizes (e.g. Cohen's *d*, Pearson's *r*), indicating how they were calculated

Our web collection on statistics for biologists contains articles on many of the points above.

Software and code

Policy information about availability of computer code

Data collection	XCalibur v.4.1
Data analysis	Xcalibur v4.1, Cutadapt v1.2.1, Sickle v1.2, metaWRAP, FastQC v0.11.9, SPAdes v3.15.2 and v3.13.3, QUAST v5.2.0, Bandage v0.8.1, BUSCO v5.4.3, rnammer v1.2, NCBI BLAST v2.11.0+, Concoct, MaxBin2, MetaBat2, CheckM v1.2.2, GTDB-Tk, Prokka v1.14.6, SSPACE Standard v3.0, Bowtie v2.4.5, Samtools v1.13-30-ga78376c, MAFFT v7.511, IQ-TREE v2.2.5, TreeViewer v2.2.0, AlphaFold v2.3.2, UCSF ChimeraX v1.7.1, custom C# scripts

For manuscripts utilizing custom algorithms or software that are central to the research but not yet described in published literature, software must be made available to editors and reviewers. We strongly encourage code deposition in a community repository (e.g. GitHub). See the Nature Portfolio guidelines for submitting code & software for further information.

Data

Policy information about availability of data

All manuscripts must include a data availability statement. This statement should provide the following information, where applicable:

- Accession codes, unique identifiers, or web links for publicly available datasets
- A description of any restrictions on data availability
- For clinical datasets or third party data, please ensure that the statement adheres to our policy

Data Availability

All raw mass spectrometry and UV-vis absorbance spectroscopy data files are freely available via UoB edata achive [https://doi.org/10.25500/edata.bham.00001196]. The P. priestleyi ANT.L61.2 genome was deposited in GenBank under the accession JBHLFI000000000 [https://www.ncbi.nlm.nih.gov/datasets/genome/GCF_053471545.1/]. The BioProject number for CCAP/SAMS strains is PRJNA1127564. The CCAP1403/21 genome was deposited under genome accession code JBHYCU000000000 [https://www.ncbi.nlm.nih.gov/datasets/genome/GCA_051861405.1/], CCAP1425/1 under JBHYDD000000000 [https://www.ncbi.nlm.nih.gov/datasets/genome/GCF_051861225.1/], CCAP1437/1 under JBHYDV000000000 [https://www.ncbi.nlm.nih.gov/datasets/genome/GCF_051860525.1/], CCAP1453/12 under JBHYEF000000000 [https://www.ncbi.nlm.nih.gov/datasets/genome/GCA_051860325.1/], CCAP1475/3 under JBIMLG000000000 [https://www.ncbi.nlm.nih.gov/datasets/genome/GCF_051859885.1/], CCAP1475/9 under JBIMLI000000000 [https://www.ncbi.nlm.nih.gov/datasets/genome/GCF_051859755.1/] and SAMS01UC under the genome accession code JBHYGB000000000 [https://www.ncbi.nlm.nih.gov/datasets/genome/GCA_051859225.1/]. Accession numbers for the genomes used in the phylogenomic analysis are provided in Table S5; raw, aligned, and trimmed sequence files are accessible from Zenodo (https://doi.org/10.5281/zenodo.17991306). Sequences used for structure prediction are listed in Table S8; sequence files, predicted structures, and confidence metrics are accessible from Zenodo (https://doi.org/10.5281/zenodo.17991306) and are provided in Supplementary Data 1.

Code Availability
C# scripts used in the methods and complete sequence alignments are available in GitHub (https://github.com/arklumpus/SoundEtAl) and archived on Zenodo (https://doi.org/10.5281/zenodo.17478265).

Research involving human participants, their data, or biological material

Policy information about studies with human participants or human data. See also policy information about sex, gender (identity/presentation), and sexual orientation and race, ethnicity and racism.

Reporting on sex and gender	N/A
Reporting on race, ethnicity, or other socially relevant groupings	N/A
Population characteristics	N/A
Recruitment	N/A
Ethics oversight	N/A

Note that full information on the approval of the study protocol must also be provided in the manuscript.

Field-specific reporting

Please select the one below that is the best fit for your research. If you are not sure, read the appropriate sections before making your selection.

☒ Life sciences ☐ Behavioural & social sciences ☐ Ecological, evolutionary & environmental sciences

For a reference copy of the document with all sections, see nature.com/documents/nr-reporting-summary-flat.pdf

Life sciences study design

All studies must disclose on these points even when the disclosure is negative.

Sample size	Cyanobacterial cultures were used to extract proteins for mass spectrometry analysis. Proteins were extracted at least 3 times from the corresponding cultures for analysis. The protein extracts were mixed and analysed over various time periods. Mass spectrometry analysis was performed repeatably on each sample, the spectra were averaged as detailed in the methods.
Data exclusions	No data was excluded
Replication	All attempts to replicate data were successful.
Randomization	This is not relevant to the study. The cyanobacterial cultures needed to be known in order to perform the experiments.
Blinding	Due to the nature of the experiments performed, blinding is not relevant to the study.

Reporting for specific materials, systems and methods

We require information from authors about some types of materials, experimental systems and methods used in many studies. Here, indicate whether each material, system or method listed is relevant to your study. If you are not sure if a list item applies to your research, read the appropriate section before selecting a response.

Materials & experimental systems

n/a	Involved in the study
☒	☐ Antibodies
☒	☐ Eukaryotic cell lines
☒	☐ Palaeontology and archaeology
☐	☒ Animals and other organisms
☒	☐ Clinical data
☒	☐ Dual use research of concern
☒	☐ Plants

Methods

n/a	Involved in the study
☒	☐ ChIP-seq
☒	☐ Flow cytometry
☒	☐ MRI-based neuroimaging

Animals and other research organisms

Policy information about studies involving animals; ARRIVE guidelines recommended for reporting animal research, and Sex and Gender in Research

Laboratory animals	The study did not involve laboratory animals. The study involve cyanobacteria. These details of the strains used are documented within the manuscript.
Wild animals	The study did not involve wild animals.
Reporting on sex	Not applicable.
Field-collected samples	No samples were collected from field.
Ethics oversight	No ethics approval was required.

Note that full information on the approval of the study protocol must also be provided in the manuscript.

Plants

Seed stocks	N/A
Novel plant genotypes	N/A
Authentication	N/A
